# Electron-Mediated Transport in Space, Astrophysical, and Laboratory Plasmas

**DOI:** 10.1007/s11214-026-01328-0

**Published:** 2026-10-05

**Authors:** Jesse T. Coburn, Maria Elena Innocenti, Daniel Verscharen, Anton Artemyev, Jasper Halekas, Wence Jiang, Matthew W. Kunz, M. J. Pueschel, Mario Riquelme, Štěpán Štverák, Lynn Wilson

**Affiliations:** 1 Mullard Space Science Laboratory, University College London, Dorking, RH5 6NT UK; 2 Laboratoire de Physique des Plasmas, CNRS, Observatoire de Paris, Sorbonne Université, Université Paris Saclay, Ecole polytechnique, Institut Polytechnique de Paris, Palaiseau, 91120 France; 3 Department of Physics and Astronomy, Ruhr-Universität Bochum, Universitätsstr. 150, Bochum, 44780 Germany; 4 Department of Earth, Planetary, and Space Sciences, University of California, Los Angeles, 90095 CA USA; 5 Department of Physics and Astronomy, University of Iowa, 414 Van Allen Hall, Iowa City, 52242 IA USA; 6 State Key Laboratory of Solar Activity and Space Weather, National Space Science Center, Chinese Academy of Sciences, Beijing, 100190 China; 7 Department of Astrophysical Sciences, Princeton University, 4 Ivy Lane, Princeton, 08540 NJ USA; 8 Princeton Plasma Physics Laboratory, PO Box 451, Princeton, 08543 NJ USA; 9 Dutch Institute for Fundamental Energy Research, De Zaale 20, Eindhoven, 5612 AJ The Netherlands; 10 Eindhoven University of Technology, PO Box 513, Eindhoven, 5600 MB The Netherlands; 11 Departamento de Física, Facultad de Ciencias Físicas y Matemáticas (FCFM), Universidad de Chile, Beauchef 850, Santiago, Chile; 12 Institute of Atmospheric Physics, The Czech Academy of Sciences, Boční II 1401/1a, Prague, Czech Republic; 13 Astronomical Institute, The Czech Academy of Sciences, Fricova 298, Ondřejov, Czech Republic; 14 Heliophysics Division, NASA Goddard Space Flight Center, Code 672, Bldg. 21, Rm. 143A, Greenbelt, 20771 USA

**Keywords:** Electron transport, Collisional, Collisionless, Heat-flux

## Abstract

Electron-mediated transport is fundamental to the thermal and dynamical evolution of plasmas across a vast range of physical environments, from the solar wind and solar atmosphere to astrophysical systems such as galaxy clusters and accretion flows, to laboratory and fusion plasmas. This article reviews the current state of understanding of electron transport, drawing together observations, simulations, and theory to contextualise key challenges and open questions. We characterise plasma environments using the Knudsen number and electron beta, and highlight that some systems fall in an intermediate regime between the collisional and collisionless limits. In the collisional limit, transport is well described by the Chapman–Enskog–Braginskii framework, yielding anisotropic heat fluxes and momentum transfer rates governed by the ratio of the electron mean free path to the electron gyroradius. In the collisionless limit, Landau-fluid closures and quasilinear wave–particle interaction theory describe heat flux regulation through resonant instabilities, particularly whistler-mode waves, which suppress parallel electron heat flux below Spitzer–Härm predictions in high-beta plasmas. We review approaches to bridging collisional and collisionless regimes, including velocity-space-dependent collision operators, moment-based interpolating closures, and data-driven methods trained on fully kinetic simulations. Comparison with solar wind observations from the Parker Solar Probe and Wind missions reveals that the current attempts at bridging collisional and collisionless closures account for only limited regions of parameter space, underscoring the need for improved models. Four central challenges are identified for future transport theory: capturing the velocity-space dependence of collisional and collisionless scattering, describing spatial transitions between transport regimes, incorporating turbulence, and developing computationally tractable effective collision operators for large-scale simulations.

## Introduction

The development of kinetic theory up to the early 1900s is largely attributed to James Clerk Maxwell, Ludwig Boltzmann, and Josiah Willard Gibbs. The utility of this theory was immediately recognised by many physicists as they sought to answer the question:“How is momentum and energy transported in a gas?” The analysis led to an elegant calculation of transport coefficients (e.g., viscosity, thermal diffusivity), which relate the higher-order moments of the particle distribution function (e.g., viscous-stress tensor, heat-flux vector) to the lower-order “fluid” moments (e.g., density, bulk velocity, and pressure). This relationship supplies a closure to the infinite chain of equations produced by taking moments of the kinetic equation, resulting in the so-called fluid equations. When the kinetic theory of ionised gases was properly posed by Anatoly Vlasov and Lev Landau, again, one of the first issues addressed by physicists was transport.

Electrons play a crucial role in transport due to the relatively small mass compared to all ions. In the collision-dominated regime, the relative smaller mass of electrons leads to shorter time scales for electron-electron and electron-ion with respect to ion-ion time scales. In the collisionless regime, the much larger thermal speed of electrons, say for a plasma with nearly equal proton and electron temperatures, largely dictates the propagation of acoustic fluctuations (see the role of electron temperature in ion acoustic fluctuations in Gary 1993; Afify et al. 2024).

Closure of the chain of moments, at the level of the heat-flux moment, is the typical goal of transport theory. The results typically express the electron heat flux, or limits on the value, as a function of the lower order moments (e.g., temperature gradient or electron beta).

In this article, we draw together simulations and observations of space, astrophysical, and laboratory plasma to contextualise the current status of our understanding of electron-mediated transport. Within this framework, we point to key challenges for future numerical and observational studies that will advance our understanding of plasma physics.

### Collisional Transport

The theory of collisional transport can be traced back to Chapman (1916), Enskog (1917), and Chapman and Cowling (1939), who performed an expansion of Boltzmann’s kinetic equation for a small ratio of the mean free path to the temperature gradient-scale of the background temperature. The expansion leads to a description of the transport of energy and momentum due to small deviations from the local thermodynamic equilibrium. Grad (1949) formalised the method by expanding the distribution function in Hermite polynomials.

The aforementioned mathematical techniques were first applied to ionised gases by Spitzer and Härm (1953), who calculated the electrical conductivity and thermal diffusion coefficients for an ionised gas. The theory was extended by Braginskii (1965, 1957) to include a subsidiary expansion in the ratio of Larmor radii to gradient length scale. Notably, Braginskii showed that collisional transport is anisotropic with respect to the magnetic field. Application of the “classical” theory to magnetically confined toroidal plasmas is summarised by Hinton and Hazeltine (1976) and Hinton (1983). The textbook by Balescu (2005) provides a comprehensive overview of transport theory in the collisonal regime.

Modern texts that follow the Chapman-Enskog-Braginskii approach can be found in the notes of Kunz (2003), and the textbook by Helander and Sigmar (2005). A modern version of the derivation that uses a projection-operator was presented by Krommes (2018a,b). The method of Grad (1949) has been extended to 22 moments by Hunana et al. (2022) (see references within) with various collision operators (Hunana 2025).

### Collisionless Transport

There are a few mathematical approaches to collisionless transport theory. One are the so-called “Landau-fluid” equations, which close the moment chain (hence, “fluid”), and capture the effect of Landau damping. The utility of these equations were recognised by Kruskal and Oberman (1958) and Rosenbluth and Rostoker (1959), and are well-presented in Russell M. Kulsrud’s book (Kulsrud 1983). The first collisionless heat-flux moment closure, based on Landau damping, was presented by Hammett and Perkins (1990). Snyder et al. (1997) extended the method to the electromagnetic guiding-center equations and Sulem and Passot (2015) have extended the approach to retain large-scale non-gyrotropic effects. For a review of the approach and a plethora of equations see Hunana et al. (2019a).

An alternative approach to understand transport in a collisionless plasma is to study the linear stability with a prescribed particle distribution function (Melrose 1986; Gary et al. 1994). In this vein, a proper understanding of collisionless transport theory likely requires an understanding of how a spectrum of electromagnetic waves interacts with the particle distribution function. In the face of significant mathematical difficulty, there has been some success in producing transport coefficients (Dum 1978b,a; Marsch 2006).

Finally, it is widely recognised that transport of momentum, energy, et cetera for many space and astrophysical plasmas of interest is determined by anomalous transport, that it is governed by an effective mean free path (Balescu 2005). The relaxation of the distribution function, in particular the particle momentum, by collisionless processes is generally referred to as *effective collisionality*. Grossly, these processes are governed by the so-called effective mean free path or effective collision frequency. Estimates of the effective mean free path or the effective collision frequency are generally much shorter than the collisional prediction (Zhuravleva et al. 2019; Kunz et al. 2020; Coburn et al. 2022). This comparison suggests that fluid equations merit more validity than one would suppose from consideration of the collisional mean free path, but it is important to bear in mind that the underlying physics is likely a resonant-diffusion process. A complete theory of the effective mean free path requires precise information on the particle distribution function and spectrum of electromagnetic waves, complicating a fluid-like closure (Dum 1978a; Shen et al. 2021).

### Electron Parameters Across Plasma Environments

**Fig. 1:**
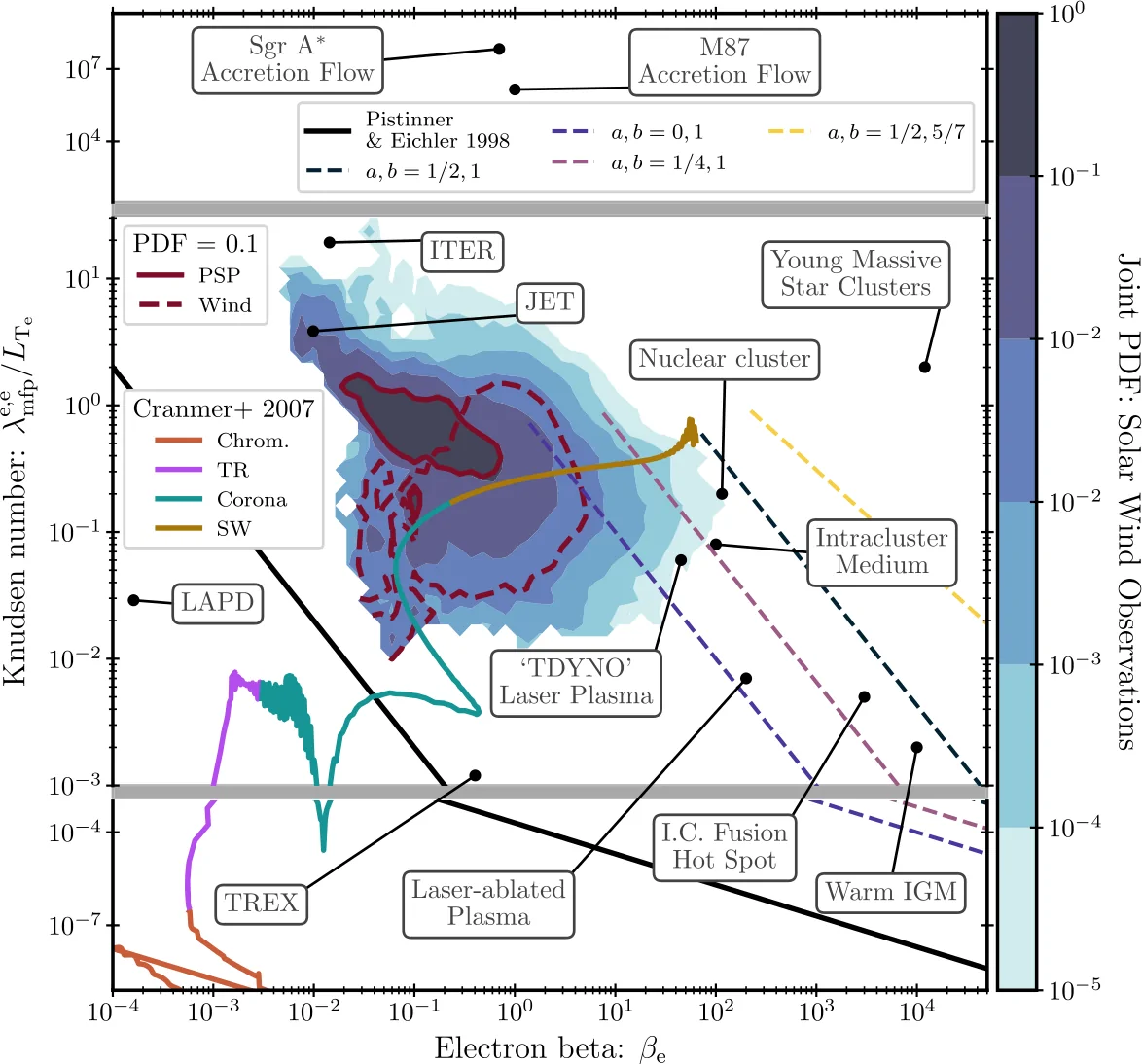
An overview of the Knudsen number Kn = $\lambda _{\mathrm{mfp}}^{\mathrm{e,e}}/L_{T_{\mathrm{e}}}$ and the electron beta $\beta _{\mathrm{e}} = 8 \pi n_{\mathrm{e}} k_{\mathrm{B}} T_{ \mathrm{e}} / B_{0}^{2}$ for a set of observations and simulations. The annotated boxes with pointers indicate parameters characteristic of laboratory and observed astrophysical systems. Citations and values for the observations appear in Table 1. The blue filled contours are solar-wind observations formed by combining the dataset recorded by Parker Solar Probe and Wind (see text for details on the observations). The red contours are ${\mathrm{PDF}} = 0.1$ for the individual spacecraft measurements: solid – Parker Solar Probe and dashed – Wind. The legend titled “Cranmer+ 2007” is a simulation of the solar atmosphere and solar wind by Cranmer et al. (2007) (see the text for details on the simulation). The abbreviated labels are “Chrom.” for chromosphere, “TR” for transition region, and “SW” for solar wind. The solid black line (Eq. (6), derived by Pistinner and Eichler 1998) divides the parameter space into collision-stablised (below the line) and whistler mode stable (above the line). The dashed coloured lines correspond to Eq. (7) for various exponents a and b, stated in the upper-right legend, and the Mach number (Ma) in Eq. (7) is set to unity. These lines delineate the relevance of kinetic instabilities in the parameter space, where the region above the lines are less stable. See the paragraph after the description of Eq. (7) for the application of these theoretical thresholds to the environments reported here

**Fig. 2:**
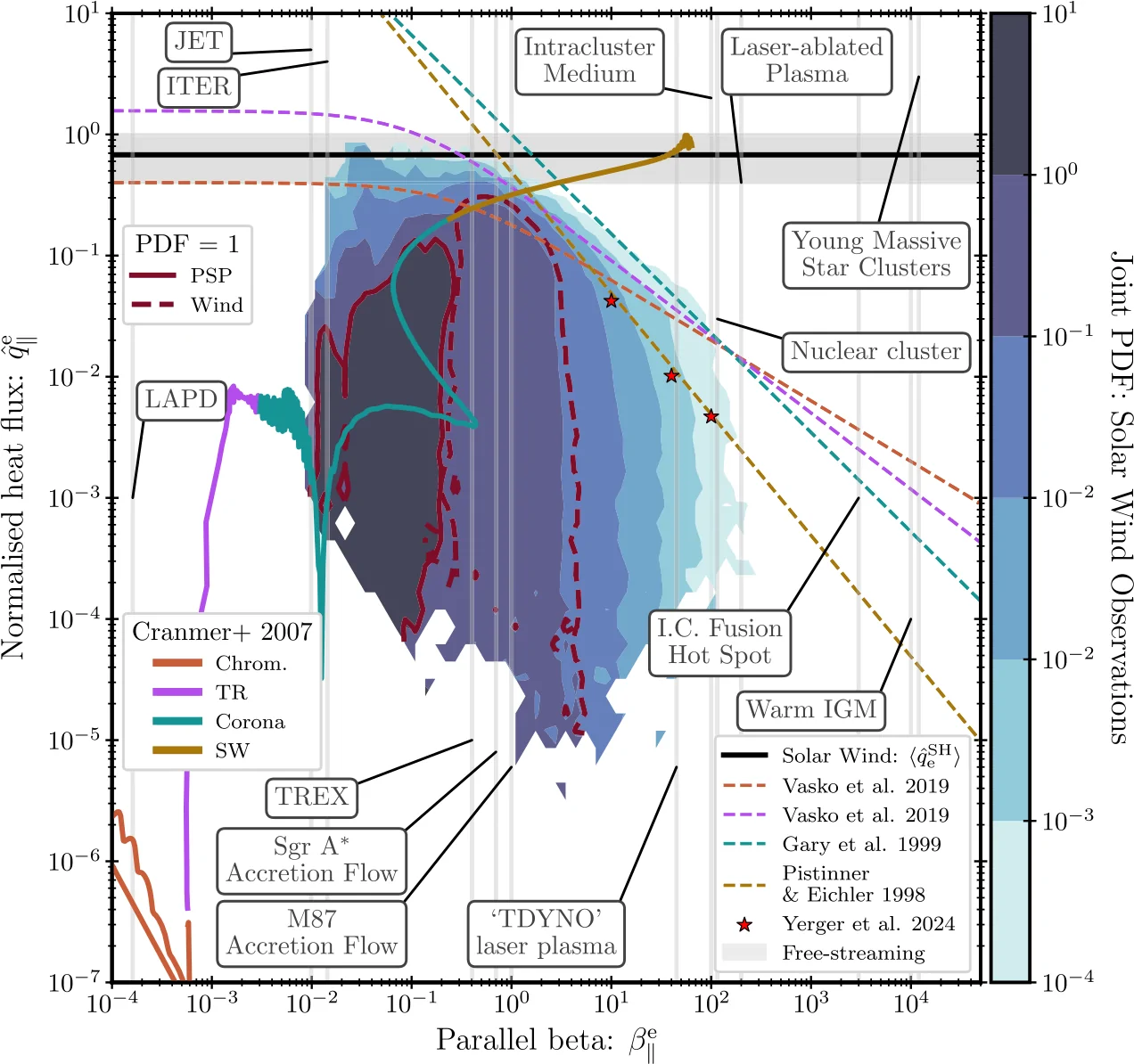
An overview of the normalised electron heat flux in space, astrophysical, and laboratory plasmas. The label titled “Cranmer+ 2007” indicates the heat flux calculated from a simulation of the solar atmosphere and solar wind by Cranmer et al. (2007), using Eqs. (17)–(22) from their paper. For more information on the simulation, see Sect. 1. The joint histograms are reported for solar-wind measurements. The red contours are the ${\mathrm{PDF}} = 0.1$ contour for the individual spacecraft measurements: solid – Parker Solar Probe and dashed – Wind. The vertical grey lines are the electron beta for various environments; reference values appear in Table 1. The black horizontal line is the average Spitzer-Härm heat flux (the $q_{\parallel}^{\mathrm{e}}$ component of Eq. (21) for $u_{\parallel}^{\mathrm{e}} = u_{\parallel}^{\mathrm{p}}$, normalised as in Eq. (8)), calculated from the solar-wind observations. See Fig. 9 for more details on the statistics. The red stars are simulation results by Yerger et al. (2025) for the electron heat flux measured in saturated state. The dashed coloured lines are collisionless heat-flux thresholds parametrised by Eq. (10); Table 3 catalogs the values. Here we plot only two thresholds from Vasko et al. (2019), which corresponds to the upper and lower limit of the beam-core drift to core thermal speed. The horizontal grey bar extends between a range of free-streaming heat flux values, the smallest of which is that of Cowie and McKee (1977)

We first introduce fundamental quantities in transport theory used for a quick determination of the dominant transport process (essentially: collisional or collisionless), and highlight their variation across a broad class of physical systems, see Fig. 1 and 2. In Sect. 2 we then return to some of those environments, and focus on environment-specific aspects of transport.

We introduce

1 $$\begin{aligned} \rho _{\mathrm{e}} = \frac{v_{\mathrm{th,e}, \perp}}{|\Omega _{\mathrm{e}}|}, \;\;\; v_{ \mathrm{th,e}} = \sqrt{ \frac{2 k_{\mathrm{B}} T_{\mathrm{e}}}{m_{\mathrm{e}}}}, \;\;\; \Omega _{\mathrm{e}} = \frac{q_{\mathrm{e}} B_{0}}{m_{\mathrm{e}} c}, \end{aligned}$$

where $\rho _{\mathrm{e}}$ is the thermal electron gyro-radius (subscript ⊥ indicates the direction perpendicular to the magnetic field), $v_{\mathrm{th,e}}$ is the electron thermal speed, and $\Omega _{\mathrm{e}}$ is the electron gyro-frequency. In addition, $k_{\mathrm{B}}$ is Boltzmann’s constant, $m_{\mathrm{e}}$ is the electron mass, $q_{\mathrm{e}}$ is the signed elementary charge, $B_{0}$ is the background magnetic field strength, and c is the speed of light. We use CGS units throughout except for the electron temperature, which is typically converted to energy via Boltzmann’s constant, then expressed in electron volts. Next,

2 $$\begin{aligned} \nu _{\mathrm{e,e}} \approx 3.6 \times 10^{-6} \, \mathrm{ln} | \Lambda _{\mathrm{e,e}} | \frac{n_{\mathrm{e}}}{\tilde{T}_{\mathrm{e}}^{3/2}} \;s^{-1} \; \mathrm{and}\; \lambda _{\mathrm{mfp}}^{\mathrm{e,e}} = \frac{v_{\mathrm{th,e}}}{\nu _{\mathrm{e,e}}} \end{aligned}$$

are the electron-electron thermal collisional frequency and the electron-electron thermal mean free path. The Coulomb logarithm is $\mathrm{ln} |\Lambda _{\mathrm{e,e}} |=\mathrm{ln} \left (\lambda _{ \mathrm{D,e}}/ |p_{0}| \right ) $, where $\lambda _{\mathrm{D,e}}$ is the electron Debye length and $p_{0}$ is the closest approach distance for which Coulomb interaction is still significant Huba (1998). Note that in the definition of Eq. (2) the electron energy $\tilde{T}_{\mathrm{e}}$ is in electron volts, and the electron number density $n_{\mathrm{e}}$ in cm^−3^.

Next, we define the correlation length of the magnetic field fluctuations ${\mathbf{b}}$ as

3 $$\begin{aligned} \ell _{\mathbf{b}} = \Bigg[ \frac{1}{|\langle{\mathbf{b}}\rangle |^{2}} \int _{- \infty}^{\infty} \mathrm{d}^{3}r \; \bigg\langle \mathrm{Tr} \big\{ { \mathbf{b}} ({\mathbf{x}}) {\mathbf{b}} ({\mathbf{x}} + {\mathbf{r}}) \big\} \bigg\rangle \Bigg]^{1/3} , \;\;\; L_{g} = \bigg(\frac{1}{g} \frac{\partial}{\partial {\mathbf{x}}} g \bigg)^{-1}, \end{aligned}$$

where $\langle ... \rangle $ denotes the ensemble average, and we assume homogeneity in ${\mathbf{x}}$. The gradient length scale is $L_{g}$ for $g = n_{\mathrm{e}} , \; |{\mathbf{u}}_{\mathrm{e}}|, \; T_{\mathrm{e}}$, where ${\mathbf{u}}_{\mathrm{e}}$ is the bulk electron velocity.

With these definitions we outline a few different regimes. We do not discuss scenarios where electromagnetic fluctuations are transported in scale by turbulence (energy cascade), dynamo, or reconnection, for example from $\ell _{\mathbf{b}}$ to $\rho _{\mathrm{e}}$ (the interested reader may refer to Verscharen et al. 2026; Jiang et al. 2026; Amano et al. 2026, all in this collection). The only gyro-scale process we discuss here is the linearly unstable case where gyro-scale fluctuations are generated, leading to quasi-linear diffusion of the distribution function. We consider the case where $L_{T_{\mathrm{e}}} \sim L_{|{\mathbf{u}}_{\mathrm{e}}|} \sim L_{n_{ \mathrm{e}}}$, which is assumed by Braginskii (1957), the latter being a result derivable from the fluid theory. The case where $L_{T_{\mathrm{e}}}$ and $L_{|{\mathbf{u}}_{\mathrm{e}}|}$ are not similar (and hence, either shear-driven or temperature-driven instabilities can be excited) has been thoroughly explored by Bott et al. (2024).

To outline regimes of electron-mediated transport we define the electron beta

4 $$\begin{aligned} \beta _{\mathrm{e}} = 8 \pi n_{\mathrm{e}} k_{\mathrm{B}} T_{ \mathrm{e}} / B_{0}^{2} \end{aligned}$$

and the Knudsen number

5 $$\begin{aligned} \mathrm{Kn} = \frac{\lambda _{\mathrm{mfp}}^{\mathrm{e,e}}}{L_{T_{\mathrm{e}}}}. \end{aligned}$$

In the fluid sense, $\beta _{\mathrm{e}}$ defines the ratio of thermal electron pressure to magnetic pressure. In regards to the kinetic regime, the square root of the $\beta _{\mathrm{e}}$ controls the ratio of the thermal speed to relevant wave phase speeds, i.e. dictating the relevance of resonant particle interactions. In the regime where Kn is large, the gradients of the macroscopic variables are too small to be consistent with the local thermodynamic equilibrium description of the plasma i.e., the description that leads to a thermal mean free path: kinetic processes govern transport. In the Kn ≪1 regime, gradients of the macroscopic variables are much larger than the length scale at which deviations from local thermodynamic equilibrium exist: transport is governed by collisional processes and so the fluid equations are valid (Braginskii 1965).

We visualize these two characteristics for a number of environments in Fig. 1, where we display an overview of the Knudsen number and of the ratio of electron thermal pressure to magnetic pressure.

In Fig. 1, we display a set of parameters relevant to astrophysical and laboratory plasmas. They are indicated with annotated boxes accompanied by dot-pointers. The values and references are recorded in Table 1 below. In the case of the International Thermonuclear Experimental Reactor (ITER) device, currently still under construction, we have used predictions (see Sips et al. 2005) that are typical of the core plasma, not the edge plasma.

**Table 1:** Reference parameters for the calculation of the quantities depicted in Fig. 1, Fig. 2, and Fig. 7. The solar wind and solar corona do not appear in the table, but appear directly in the Figures and are explained in the text

Record of the values plotted in Fig. 1, Fig. 2, and Fig. 7								
Environment	*T̃* [keV]	$n_{\mathrm{e}}$ [cm^−3^]	$B_{0}$ [G]	$L_{T_{\mathrm{e}}}$	$\lambda _{\mathrm{mfp}}^{\mathrm{e,e}}$ [cm]	$\rho _{\mathrm{e}}$ [cm]	$\beta _{\mathrm{e}}$	Kn
Sgr A Accretion Flow (Event Horizon Telescope Collaboration et al. 2022)	2 × 10^4^	1 × 10^6^	30	3 × 10^12^ cm	2 × 10^20^	400	0.7	7 × 10^7^
M87 Accretion Flow (Event Horizon Telescope Collaboration et al. 2019)	2 × 10^4^	3 × 10^4^	5	5 × 10^15^ cm	7 × 10^21^	3 × 10^3^	0.7	1.4 × 10^6^
Young Massive Star Clusters (Badmaev et al. 2022)	3	1	3 × 10^−6^	3 × 10^18^ cm	6 × 10^18^	5 × 10^7^	12,000	2
Nuclear Cluster (Ressler et al. 2018, 2020)	2.6	100	3 × 10^−4^	3 × 10^17^ cm	6 × 10^16^	5 × 10^5^	115	0.2
Intracluster Medium (Simionescu et al. 2011; Bott et al. 2024)	5	10^3^	2 × 10^−7^	100 kpc	2 × 10^22^	10^9^	100	0.08
Warm Intergalactic Medium (Bott et al. 2024)	10^2^	10^−5^	10^−8^	3 × 10^24^ cm	6 × 10^21^	6 × 10^9^	10^4^	2 × 10^−3^
Internal Confinement Fusion Hot Spot (Abu-Shawareb et al. 2022; Sadler et al. 2022)	10^4^	2 × 10^25^	8 × 10^7^	5 × 10^−3^ cm	2.5 × 10^−7^	5 × 10^−8^	3 × 10^3^	5 × 10^−3^
‘TDYNO’ Laser Plasma (Bott et al. 2024)	1	5 × 10^20^	10^6^	10^−2^ cm	6 × 10^−4^	6 × 10^−5^	45	6 × 10^−2^
Laser-ablated Plasma (Bott et al. 2024)	1	4 × 10^21^	10^6^	10^−2^ cm	7 × 10^−5^	2.8 × 10^−5^	200	7 × 10^−3^
International Thermonuclear Experimental Reactor (ITER) (Sips et al. 2005)	10	10^14^	5.3 × 10^4^	620 cm	1.2 × 10^4^	6 × 10^−5^	0.014	20
Joint European Torus (JET) (Federici et al. 2001)	3	10^14^	3.5 10^4^	300 cm	10^3^	5.3 × 10^−5^	0.01	4
Large Plasma Device (LAPD) (Perks et al. 2022)	4 × 10^−3^	10^12^	100	10 cm	0.3	7 × 10^−5^	10^−4^ − 10^−2^	3 × 10^−2^
Terrestrial Reconnection Experiment (TREX) (Forest et al. 2015)	10^−2^	10^13^	100	150 cm	0.2	10^−3^	0.4	10^−3^

The filled contour is a joint probability density function (PDF) of solar-wind observations measured by two spacecraft: Parker Solar Probe (PSP) (Fox et al. 2016) and Wind (Ogilvie and Desch 1997). We show the combined distribution with a blue shaded contour because they significantly overlap one another. To get a sense of the individual distributions, we show the ${\mathrm{PDF}} = 10^{-1}$ contour from the separated distributions as different red line styles; see the legend for details. The major difference between the two spacecraft are electron conditions across the differing radial distances to the Sun: PSP observations range from $11.4~{\mathrm{R}}_{\mathrm{Sun}}$ to $53.8~{\mathrm{R}}_{\mathrm{Sun}}$, while the Wind observations shown here were primarily taken near $216~{\mathrm{R}}_{\mathrm{Sun}}$, since it is stationed at the L1 Lagrange point near to Earth. Here, ${\mathrm{R}}_{\mathrm{Sun}}$ is the solar radius, approximately $6.975 \times 10^{5}~{\mathrm{km}}$. In astronomical units ($1~{ \mathrm{au}} = 1.496 \times 10^{8}~{\mathrm{km}}$), PSP observations span $0.053~{\mathrm{au}}$ – $0.24~{\mathrm{au}}$ and Wind’s radial distance from the Sun is approximately $1~{\mathrm{au}}$. In brief, closer to the Sun, plasma parameters such as the magnetic field strength, density, and temperature are greater, while the plasma beta is smaller (Matteini et al. 2013). The calculation of the Knudsen number requires measurement of the temperature gradient scale. For the solar wind, the typical method is to measure the electron temperature over a span of radial distances R and assume that it follows a power law $T_{\mathrm{e}} \propto R^{\alpha}$. We estimate the temperature gradient with this method, by fitting PSP and Wind temperatures as a function of radial distance. We find a power-law exponent of $\alpha = -0.531 \pm 10^{-3}$. The most appropriate method to estimate the temperature gradient scale for solar wind electrons is a subject of debate and is discussed in Sect. 2.1.

The multi-colour solid line labeled in the legend “Cranmer+ 2007” is the “equatorial” (i.e. in the ecliptic plane where the Earth orbits the Sun) parameterisation of a simulation performed by Cranmer et al. (2007). The simulation models the solar atmosphere and solar wind out of a coronal hole as a radial flux tube and solves an appropriate set of single-fluid plasma equations on a prescribed background magnetic field. The simulation is driven by acoustic waves in the chromosphere, which provide heat, and by Alfvén waves in the corona, generated by a wave reflection parameter, which provide acceleration. The latter effect and pressure gradient (Parker 1958) accelerate the plasma and form the solar wind. The electron heat flux is closed with a function that asymptotes between Braginskii’s electron conductivity and Hollweg’s free-streaming heat flux, based on the ratio of the local wind expansion rate and the collision frequency. The simulation is well regarded for its consistency with coronal electron temperature and density measurements by Landi (2008).

The simulation results by Cranmer et al. (2007) permit us to compute the local temperature gradient and therefore provide considerable understanding of how the Knudsen number changes for realistic profiles of the electron density, temperature, and magnetic field. The color distinctions are based on physically distinct regions of the solar atmosphere (e.g. density and temperature, which set spectral emission characteristics and collisionality). The chromosphere (“Chrom.”) is shown as a red solid line that extends up to $0.0087~{\mathrm{R}}_{\mathrm{Sun}}$. The lower boundary of the chromosphere is the photosphere, which is not shown in Fig. 1 but is in the lower left hand corner. The transition region (“TR”) is shown as a violet line in Fig. 1. For this particular simulation the TR spans radial distances $0.0087~{\mathrm{R}}_{\mathrm{Sun}}$–$0.014~{\mathrm{R}}_{\mathrm{Sun}}$, but this is not well constrained by numerical models and observations. This sharp increase in the Knudsen number is primarily due to the rapid rise in temperature, although there persists a longstanding debate on the relative importance of various heating mechanisms, mainly magnetic reconnection and waves/turbulence (Cranmer and Winebarger 2019). The simulation by Cranmer et al. (2007) relies on waves and turbulence to heat and accelerate the plasma.

The corona (shown as a teal line) traverses a few orders of magnitude in both the Knudsen number and the electron beta, spanning $0.014~{\mathrm{R}}_{\mathrm{Sun}}$– $19~{\mathrm{R}}_{\mathrm{Sun}}$. In this region, ionisation and recombination continue to occur, but the plasma is fully ionised. Finally, the solar wind (“SW” in Fig. 1) is traced by the yellow line, which spans from $19~{\mathrm{R}}_{\mathrm{Sun}}$ to the outer boundary of the simulation: $1200~{\mathrm{R}}_{\mathrm{Sun}}$. In reality, the solar wind spans to the edge of the heliosphere, approximately 80– $100~{\mathrm{au}}$, but within a few au from the Sun, interstellar neutrals drastically affect the out-flowing plasma (Burlaga et al. 1996). In the corona and the solar wind, the fluid treatment of the plasma by Cranmer et al. (2007) breaks down, and so we do not expect exact correspondence between the solar wind observations and the model.

We consider the simulation by Cranmer et al. (2007) to be representative of time-stationary (“background”) parameters of the solar atmosphere and solar wind. The solar wind observations shown in Fig. 1 display a variety of solar wind “background” conditions and “fluctuations” from the background. It is therefore natural to expect that any of the values listed for the environments in Table 1 will vary significantly when observed at higher resolution, as it is the case for the solar wind. In addition, it is likely that many of these environments require a sophisticated treatment of transport, one that describes both the collisional and collisionless regimes. We explore attempts to model this in full detail in Sect. 3.3.

### The Stability of Electrons in Weakly Collisional Plasma

To briefly discuss electron stability to a number of different kinetic instabilities in the weakly collisional regime, we include parameterised instability thresholds in Fig. 1. The solid black line indicates the threshold in the Knudsen number

6 $$\begin{aligned} \mathrm{Kn} = \frac{2 \times 10^{-4}}{\beta _{\mathrm{e}}}, \end{aligned}$$

derived by Pistinner and Eichler (1998), which divides the parameter space into regions where regulation of the electron heat flux is governed by collisions (below the line) and interaction with whistler waves (above).

The dashed coloured lines are derived by Bott et al. (2024). The lines distinguish the regions of Kn – $\beta _{\mathrm{e}}$ space in which a weakly collisional plasma becomes subject to a specific kinetic instability. The unstable region lies in the upper right region relative to the threshold lines. See Table 2 of Bott et al. (2024) and surrounding discussion for more details on these stability thresholds. These lines are parameterised by

7 $$\begin{aligned} \mathrm{Kn} = \bigg(\frac{m_{\mathrm{p}}}{m_{\mathrm{e}}} \bigg)^{a} \frac{1}{\beta _{\mathrm{e}}^{b}} \frac{1}{\mathrm{Ma}} \end{aligned}$$

where we set the Mach number Ma $=|{\mathbf{u}}^{\mathrm{p}}|/v_{\mathrm{th,p}} = 1$, ${\mathbf{u}}^{\mathrm{p}}$ is the bulk proton velocity, $v_{\mathrm{th,p}} = \sqrt{2 k_{\mathrm{B}} T_{\mathrm{p}}/ m_{ \mathrm{p}}}$ is the proton thermal velocity, $T_{\mathrm{p}}$ is the proton temperature and $m_{\mathrm{p}}$ is the proton mass. This choice is consistent with the assumptions of Braginskii (1965) that ${\mathrm{Ma}} \sim 1$, but it is not consistent with the solar wind, where the protons are supersonic ($\mathrm{Ma} \gg 1$) and the electrons, observed to have $T_{\mathrm{p}} \sim T_{\mathrm{e}}$ and negligible current $|{\mathbf{u}}^{\mathrm{e}}| \approx |{\mathbf{u}}^{\mathrm{p}}|$, are subsonic. The parameters a and b in Eq. (7) are found in the legend of Fig. 1. The thresholds a=1/4,b=1 are associated with kinetic instabilities generated by electron heat flux, while the remaining thresholds are associated with shear in the bulk velocity. Due to the large class of instabilities, we direct the reader to Bott et al. (2024) (in particular Table 2) for a thorough derivation and explanation. These instability thresholds are described and discussed in more detail in Sect. 3.3.

The applicability of the thresholds expressed by Eqs. (6) and (7) to the various regimes we consider is worth questioning, as some assumptions are violated or have yet to be addressed by observations. The incorporation of these thresholds into our figures is meant to act as a guide to the underlying physics and to remind the reader that weakly collisional plasmas at moderate and high $\beta _{\mathrm{e}}$ are subject to kinetic instabilities that are likely to dominate transport phenomena. The relevance of the thresholds and resulting evolution of the plasma requires synthesis of numerical modeling and observations. Active research questions include: what is the dominant instability, how is it triggered, and what the details are of the saturated state? The articles by Komarov et al. (2018), Kuzichev et al. (2019), Micera et al. (2021), Kuzichev et al. (2023), Bott et al. (2024), Yerger et al. (2025) can be referred for more information.

Figure 2 has been constructed to aid our understanding of what limits the electron heat flux in space, astrophysical, and laboratory plasma. It depicts in the vertical axis the normalized parallel heat flux, in the horizontal axis the parallel plasma beta. This is a useful representation given the role of electromagnetic instabilities in heat flux regulation, see later Eq. (10). Since we do not have direct measurements of the electron heat flux across the environments listed in Table 1, the values corresponding to the different systems are plotted as vertical lines, positioned at the electron beta. The vertical axis is the normalised parallel electron heat flux

8 $$\begin{aligned} \hat{q}^{\mathrm{e}}_{\parallel} = \frac{q^{\mathrm{e}}_{\parallel}}{q_{\mathrm{sat}}} , \hspace{1cm} q_{\mathrm{sat}} = \frac{3}{2} n_{\mathrm{e}} k_{\mathrm{B}} T_{ \mathrm{e}} v_{\mathrm{th, e}} , \end{aligned}$$

where $q^{\mathrm{e}}_{\parallel}$ is the parallel electron heat flux – the parallel flow of parallel heat, or the parallel component of the relevant gyrotropic heat flux vector. The normalisation by the free-streaming “saturated” heat flux $q_{\mathrm{sat}}$ is useful for classifying kinetic instabilities and is proportional to various estimates of the microphysically regulated heat flux to within an order-unity coefficient. The saturated heat flux was introduced by Hollweg (1974) and Cowie and McKee (1977) as the maximum heat flux, being the scenario where a collisionless and nearly Maxwellian plasma is subjected to an infinite temperature gradient. Some authors define the saturated heat flux with a factor of 0.4 out front, so we indicate this with as a grey horizontal bar in Fig. 2 that spans [0.4,1.0].

In Fig. 2, similarly to Fig. 1, solar wind observations are combined, and the contours ${\mathrm{PDF}} = 1$ of the individual joint PDFs are shown, together with results from the simulation by Cranmer et al. (2007). We calculate the PSP electron heat flux values using data taken from the SWEAP and FIELDS experiments (Kasper et al. 2016; Bale et al. 2016), using the techniques described by Halekas et al. (2021). The electron Velocity Distribution Functions (VDFs) measured by the SPAN-Electron sensors (Whittlesey et al. 2020) are analysed in the plasma frame (as determined from the ion bulk velocity measured by the SPAN-Ion sensor (Livi et al. 2022)), in magnetic-field-aligned coordinates. To avoid contamination by secondary electrons from spacecraft and instrument surfaces at low energies, we fit the electron core population to a drifting bi-Maxwellian to determine the core heat flux (typically sunward). The residual to this fit provides the suprathermal velocity distribution, which we integrate over to determine the suprathermal moments. The total electron heat flux, in the plasma frame, then represents the sum of the core and suprathermal heat flux.

The electron measurements made by Wind follow the method described by Salem et al. (2023). The three-dimensional electron VDF is measured with top-hat symmetrical spherical section electrostatic analyzers (Lin et al. 1995). The electron dataset is calibrated with independent measurements of the electron density and spacecraft potential by the WAVES experiment (Bougeret et al. 1995), following the method of Salem et al. (2001). In addition, the magnetic field, as measured by the onboard magnetometer (Lepping et al. 1995) and the proton moments, measured by the Solar Wind Experiment (Ogilvie et al. 1995), are used in calibration and to transform the measurements into the plasma frame. In brief, a sophisticated model of solar wind electrons is fitted to solar wind observations, from which the moments can be obtained for densities, temperatures, and heat fluxes. The selection criteria were first discussed by Salem et al. (2023) and later refined by Wilson et al. (2018) and Wilson et al. (2023).

The Cranmer et al. (2007) simulations for the solar atmosphere and solar wind assume that the electron heat flux bridges between the Spitzer-Härm result in the collisional regime and to a form of the saturation heat flux in the collisionless regime, which is typically called the free-streaming heat flux. The free-streaming result was derived by Hollweg (1974), it is appropriate to the solar wind where the solar wind plasma is assumed to be quasi-Maxwellian with zero-current and the protons and electrons have similar temperatures. The bridge between the two regimes is discussed in more detail in Sect. 3.3.

Next, we plot the average solar-wind Spitzer–Härm heat flux

9 $$\begin{aligned} q^{\mathrm{SH}}_{\parallel \mathrm{e}} = - \kappa _{\parallel , T}^{ \mathrm{e}} \frac{\partial}{\partial x_{\parallel}} T_{\mathrm{e}} , \hspace{0.5cm} \kappa _{\parallel , T}^{\mathrm{e}} = \hat{\kappa}_{\parallel , T}^{ \mathrm{e}} \frac{p_{\mathrm{e}}}{m_{\mathrm{e}} \nu _{\mathrm{e,e}}} \end{aligned}$$

where $\kappa _{\parallel , T}^{\mathrm{e}}$ is the parallel electron conductivity and $\hat{\kappa}_{\parallel , T}^{\mathrm{e}} $ is an order unity coefficient calculated from kinetic theory. This is discussed in better detail in Sect. 3.1. When normalised to the saturated value it is $\hat{q}^{\mathrm{SH}}_{\mathrm{e}} \approx \mathrm{Kn}$. The standard deviation of $\hat{q}^{\mathrm{SH}}_{\mathrm{e}}$ is larger than 1 so we do not indicate it in Fig. 2. Notice that $q_{\mathrm{sat}} \approx \langle \hat{q}^{\mathrm{SH}}_{\mathrm{e}} \rangle $ for the solar wind, which is typically larger than $\hat{q}^{\mathrm{e}}_{\parallel}$, but not by more than one standard deviation. This similarity has made it difficult to confirm which mechanisms determine the solar-wind electron heat flux: collisions or wave-particle instabilities (cf. Bale et al. 2013; Halekas et al. 2021).

We plot a set of instability thresholds as dashed-colour lines, see the bottom right legend. They are parametrised by (Vasko et al. 2019)

10 $$\begin{aligned} \hat{q}^{\mathrm{e}}_{\mathrm{inst}}(S_{q}, a_{q}, \alpha _{q}) = \frac{1}{q_{\mathrm{sat}}} \frac{S_{q}}{(\beta _{\mathrm{e}} + a_{q})^{\alpha _{q}}} . \end{aligned}$$

The parameters $(S_{q}, a_{q}, \alpha _{q})$ depend upon the shape of the assumed model distribution function. We further explore these instability thresholds in Sect. 3.2.2. In general, these thresholds quantify the stability of the electron distribution function to the cyclotron-resonant whistler waves. The $1/\beta _{\mathrm{e}}$ scaling arises from considering the cyclotron interaction between electrons and whistler waves. In the regime $\Omega _{\mathrm{p}} \ll \omega _{\mathrm{whist}} \ll |\Omega _{ \mathrm{e}}|$, whistler waves have the dispersion relation $\omega _{\mathrm{whist}} \approx k_{\parallel}^{2} d_{\mathrm{e}} | \Omega _{\mathrm{e}}| $. If we assume that the distribution function is approximately isotropic $\beta ^{\mathrm{e}}_{\perp} \approx \beta ^{\mathrm{e}}_{\parallel}$ and that the wavenumber $k_{\parallel} \approx 1/\rho _{\mathrm{e}}$ dominates the growth rate, then the resonant velocity is $v^{\mathrm{res}}_{\parallel} = v_{\mathrm{th,e}}(p + \beta ^{-1}_{ \mathrm{e}})$ where p is the order of the cyclotron resonance. That $\hat{q}_{\mathrm{inst}}^{\mathrm{e}}$ is proportional to $1/\beta _{\mathrm{e}}$ was pointed out by Gary et al. (1975) and Pistinner and Eichler (1998). The parameters that lead to the exact form of Eq. (10) depend on the form of the electron velocity distribution function e.g. shape of the electron beam. Recently, considerable progress on the inhibition of the electron heat flux in the collisionless non-linear regime has been made by Roberg-Clark et al. (2018a), Komarov et al. (2018), Kuzichev et al. (2019), Drake et al. (2021), Kuzichev et al. (2023), Yerger et al. (2025).

The high-$\beta _{\mathrm{e}}$ regime of the solar wind provides the best empirical evidence we have for wave-particle suppression of the electron heat flux. Kinetic models (Vocks et al. 2005; Jeong et al. 2020; Tang et al. 2020), including fully kinetic simulations run with solar wind parameters (Kuzichev et al. 2019; Micera et al. 2020, 2021; Kuzichev et al. 2023; Micera et al. 2025) support this model. Additional theoretical evidence comes from Yerger et al. (2025), who analysed kinetic simulations of moderately high-$\beta _{\mathrm{e}}$, collisionless, magnetised plasma subject to a background temperature gradient. The plasma is unstable to an electron heat-flux whistler instability that subsequently modifies the electron heat transport. Figure 2 shows results from their simulation as red stars.

## Details on the Plasma Environments

In this Section, we provide further heat transport information that relates specifically to several space, astrophysical and laboratory environments. The interested reader may refer to Wicks et al. (2027, this collection) for a review of electron physics across the universe.

### Solar Wind

Parker (1958) showed that coronal temperatures of 1 million K inevitably produce the outflow of ionised gas which we call the solar wind. Among the many assumptions and details, the energy budget is closed by the thermal conductivity given by Chapman (1954).^1^ Electrons in the solar atmosphere mediate thermal transport out to large radial distance from the Sun, preventing a hydrostatic equilibrium.

Jockers (1970) also proposed a theory of solar-wind acceleration where electrons transport plays a key role. In the two-fluid (proton - electron) picture, the electrons, being less massive, are more easily accelerated. This induces an electric field that provides an additional acceleration on the near-Sun ions. These so-called exospheric models can be derived from fluid or kinetic equations, and provide predictions for the shape of the electron distribution function heliosphere (Lemaire and Scherer 1973; Meyer-Vernet and Issautier 1998; Maksimovic et al. 2001; Zouganelis et al. 2005). The critical component of these models is to recognise that the Kn transitions from below to above unity near to the Sun: transport transitions from collision-dominated to collisionless, making it a complex transport process. Recent Parker Solar Probe and Solar Orbiter observations have characterized the radially-dependent interplanetary electric potential predicted by exospheric models through its signatures on the electron velocity distribution function (Halekas et al. 2020; Berčič et al. 2021a,b).

The solar wind is one of the few naturally occurring plasmas that can be directly sampled, a so-called natural laboratory, see reviews by Marsch (2006), Bruno and Carbone (2013), Verscharen et al. (2019b). As seen in Fig. 1, the solar wind Kn and $\beta _{\mathrm{e}}$ span a few orders of magnitude, both centered around unity. In principle, the solar wind should be an excellent environment to test theories of electron transport, both collisional and collisionless.

Hollweg (1974) investigated thermal transport of electrons in the solar wind and showed that without collisions, the negligible current is sufficient to produce a relation

11 $$ q^{\mathrm{Holl}}_{\mathrm{e}} = \alpha _{\mathrm{Holl}} n_{ \mathrm{e}} k_{\mathrm{B}} T_{\mathrm{e}} |{\mathbf{u}}_{\mathrm{p}}| , $$

where $\alpha _{\mathrm{Holl}}$ is a dimensionless parameter of order of unity (determined by the exact shape of the distribution function) and ${\mathbf{u}}_{\mathrm{p}}$ is the solar wind velocity. Hollweg (1974) also points out that kinetic instabilities should also be effective at reducing the electron heat flux, and so Eq. (11) is a rough upper estimate. In Sect. 3.2, we discuss kinetic instabilities that act to reduce the total electron heat flux.

Indeed, solar-wind electrons are typically observed to have a few persistent non-Maxwellian features. The solar-wind heat flux is mostly carried by suprathermal electrons, and in particular, by the so-called strahl electrons, a highly anisotropic, field-aligned electron population moving in the anti-sunward direction (Feldman et al. 1975; Pilipp et al. 1987; Bale et al. 2013; Halekas et al. 2020). In addition, there is the halo population, which is a quasi-isotropic suprathermal feature that follows a power law with particle energy (Štverák et al. 2015).

Following the chronology of solar wind electron transport studies, Montgomery et al. (1968) showed that the electron heat flux is consistent with the collisional transport theory of Spitzer and Härm (1953) (see Eq. (9)) with measurements by the Vela 4 spacecraft. This result was then challenged by measurements spanning different heliocentric distances, from 0.3 (Helios) to several (Ulysses) au, where the Spitzer-Härm prediction appeared insufficient to justify the observed heat flux evolution with distance (Scime et al. 1994). More recently, Salem et al. (2003), Bale et al. (2013) showed, with observations of solar-wind electron heat flux at 1 au by the Wind spacecraft, a transition between the Spitzer-Härm result and the collisionless regime near Knudsen numbers of order unity. Landi et al. (2014), working in a regime compatible with Bale et al. (2013), has shown with numerical modeling that the heat flux is still not collision dominated for Knudsen numbers down to about Kn∼0.02, and showed that the heat flux behavior and its radial profile is rather consistent with the free-streaming collisionless form of Eq. (11).

The correct form of the electron heat flux is hard to assess based on in situ observations. First, substantial difficulties in deriving the spatial gradients of the heat flux or the temperature (cf., Eq. (14)) are caused by the single-point nature of the spacecraft measurements due to spatiotemporal variability. In fact, measured temperature gradients in the solar wind from numerous electron measurements span a wide range from nearly adiabatic to almost isothermal radial profiles for different solar wind streams and regimes (see, e.g., Ogilvie and Scudder 1978; Feldman et al. 1979; Pilipp et al. 1990; Scime et al. 1994; Issautier et al. 1998; Maksimovic et al. 2000). Second, the two heat-flux models given by Eqs. (9) and (11) are very close to estimates of the heat flux based on measured solar-wind properties (see Fig. 2). Equivalently, none of the observed heat-flux gradients (see, e.g., Pilipp et al. 1990; Scime et al. 1994; Štverák et al. 2015; Halekas et al. 2020), are consistent with theoretical collisional or free-streaming profiles (Scime et al. 1994).

More in depth studies of the energy balance provide further constraints on the electron heat flux. In the simplest form, the local energy balance in the spherically symmetric form can be written as

12 $$ \frac{\partial T_{\mathrm{e}}}{\partial R} + \frac{4}{3} \frac{T_{\mathrm{e}}}{R} + \frac{1}{n_{\mathrm{e}} k_{\mathrm{B}} u R^{2}} \frac{\partial }{\partial R} \left ( q_{\mathrm{e}} R^{2}\right )= 0, $$

where R is the radial coordinate which we have assumed to be time-stationary, collisionless, and without any heat sources. The radial electron temperature is governed by the heat flux, see Hubert et al. (2023). With observations by Ulysses, Scime et al. (1994) was able to provide conclusive evidence that some processes regulate the heat flux beyond collisions: observations point to kinetic instabilities (Gary et al. 1975, 1994).

Cluster II, Helios and PSP observations by Lacombe et al. (2014), Jagarlamudi et al. (2020), Cattell et al. (2021) show that whistler instabilities can scatter strahl electrons, thus reducing the electron heat flux. Kinetic models, including fully kinetic simulations run with solar wind parameters, show that instabilities in the whistler family can constrain heat flux in the solar wind by scattering strahl electrons, generating the halo electron population in the process (Vocks et al. 2005; Jeong et al. 2020; Tang et al. 2020; Kuzichev et al. 2019; Vasko et al. 2019; Verscharen et al. 2019a; Vasko et al. 2020; Micera et al. 2020, 2021, 2025). In particular, Micera et al. (2021) run fully kinetic expanding box simulations (Innocenti et al. 2019) initialized with parameters taken from PSP Encounter 1 observations. They show that observed, stable electron VDFs are driven unstable by the variation of background plasma parameters associated with solar wind plasma expansion. An oblique whistler heat flux instability ensues, and scatters strahl electrons into the halo. We further explore these topics in Sect. 3.2.

Observations of non-adiabatic temperature profiles motivate the importance of empirically quantifying the energy balance to test also for the existence of other possible physical mechanisms responsible for an external heating. To date, few studies have addressed the non-adiabatic behavior of the electron temperature (Cranmer et al. 2009; Štverák et al. 2015; Abraham et al. 2022). Notwithstanding the substantial difficulties in deriving the temperature and heat-flux gradients from single-point measurements, the observed energy imbalance suggests other heating/cooling processes are active. A natural candidate is the turbulence observed throughout the solar wind (Bandyopadhyay et al. 2023a).^2^

### Solar Atmosphere

The solar atmosphere exhibits multiple plasma regimes where electron transport plays a critical role in – and lead to many observables of – the underlying physical processes (West et al. 2023). The scattering of photospheric light by electrons, known as Thomson scattering, produces substantial emission throughout the solar atmosphere, illuminating the magnetic structures of the corona (Habbal et al. 2010).

Starting at the photospheric base, initial ionisation of the plasma is determined by regions where the electron energy exceeds the first ionisation potential; the process critically depends upon electron transport. Material is then transported through the chromosphere and transition region ($0 \leq R \leq 0.003 R_{\mathrm{Sun}}$) by a set of competing processes: heating by waves, collisions with neutrals, and the force of gravity (Geiss et al. 1970; Schwadron et al. 1999; Laming 2015). This processes ultimately determines the heavy ions abundances in the soalr wind.

Moving out radially from the Sun’s surface the density of the solar atmosphere decreases by ${\sim}10^{8}$ while the electron temperature increases by ${\sim}10^{3}$, encompassing the famous sharp rise in temperature known as the transition region; explaining this sharp rise constitutes a notable unsolved electron-transport problem (Cranmer and Winebarger 2019; Barbieri et al. 2024). The problem is thought to be a consequence of transport of electrons across the transition region, for example, the ability of the electron beam and kappa-like tail of the electron distribution to form (Scudder and Olbert 1979; Scudder 1992).

The high density and temperature of the solar atmosphere permit various atomic electron transitions, typically spontaneous emission following resonant or collisional excitation with electrons, with their occurrence proportional to $n_{\mathrm{e}}$ and $n_{\mathrm{e}}^{2}$, respectively (Zanna and Mason 2018). In the innermost part of the solar atmosphere, collisional excitation is a ion-electron process, which is therefore sensitive to the electron temperature and density, hence transport. This provides solar physicists with the ability to reliably associate observed emission intensities of spectral lines with electron temperatures.

Spacecraft in the solar wind make direct measurements of the ion abundance, which is observed to correlate well with solar-wind speed. The relative ion abundances serve as a key diagnostic to understand solar-wind acceleration processes and to identify source regions of solar-wind plasma. Ultimately, the electrons have to be heated to ionise the neutrals, so the charge state of the heavy ions can be used to infer electron temperature (Ko et al. 1997; Laming et al. 2019). Further ionization and recombination of ions occur further out in the solar atmosphere in the corona, due to the increase of the electron temperature (Hundhausen et al. 1968). Both processes are proportional to the density and complicated functions of temperature. The ions that escape the gravitational potential of the Sun and form the solar wind expand into interplanetary space. As the coronal plasma expands, the density decreases; eventually the recombination and ionisation become negligible and the ion charge state *freezes in* (Landi et al. 2012). The ionisation state of solar-wind ions is measurable and therefore serves as a diagnostic of the coronal electron temperature (Rivera et al. 2022). The exact charge-state distribution, e.g. the density of $C^{4+}$, $C^{5+}$ and $C^{6+}$, where C stands for carbon and the superscript for the ionization state, which are determined by the radial dependence of the ion velocity, electron temperature and electron density. Transport mediated by electrons becomes important to understand the coronal processes.

Despite the fairly low Knudsen number of the solar atmosphere (see Fig. 1) the line-widths of electromagnetic spectra suggest that electrons are not in local thermodynamic equilibrium (Brown et al. 2008; Brooks and Warren 2016; Pant and Van Doorsselaere 2020). The prediction of these line-widths by heating models has gained a lot of attention for their ability to address the unsolved coronal heating problem, including parametrization of the electron heat flux (Patsourakos and Klimchuk 2005; Bradshaw and Cargill 2006). Therefore, a successful description of the electron heat flux, temperature, and the transport coefficients in a partially ionized plasma is an essential ingredient in models of the solar atmosphere and crucial for interpreting the observations.

### Astrophysical Plasmas

Electron heat conduction plays a critical role in the evolution of a wide range of astrophysical systems and processes, including supernova remnants (Steinwandel et al. 2020; Cui and Cox 1992), planetary nebulae (Steffen et al. 2008; Sandin et al. 2012, 2016), the formation of galaxies (Fabian et al. 2002; Choi et al. 2022), the thermodynamic evolution of the intracluster medium (ICM) in galaxy clusters (Binney and Cowie 1981), and others. In the case of the ICM, conduction may play a crucial role in preventing radiative cooling, which, if left unrestrained, may lead to significant mass accretion rates of up to thousands of solar masses per year. Such accretion rates are much larger than those inferred from X-ray observations of clusters (e.g., Peterson et al. 2003): understanding which heat transport process contrasts the expected radiative cooling constitutes the *cooling flow problem* (Fabian and Nulsen 1977).

Thermal conduction will lower accretion rates (Zakamska and Narayan 2003; Kim and Narayan 2003), but a balance with Bremsstrahlung cooling is thermally unstable. Other processes like streaming cosmic rays and active galactic nuclei (AGN) activity are also promising heating candidates to balance the ICM cooling. AGN activity is also expected to be significantly affected by thermal conduction (Fabian et al. 2005; Yang and Reynolds 2016; Zweibel et al. 2018), underscoring the key role of heat transport in the thermodynamic evolution of the ICM.

Observed temperature gradients in galaxy clusters show that heat conduction in these environments is suppressed by factors of ${\sim}0.1$ to ${\sim}10^{-2}$ relative to Spitzer values (Ettori and Fabian 2000; Markevitch et al. 2003). This strongly suggests that heat transport in clusters is affected by physical processes that go beyond classical Coulomb collisions. One possible explanation is that in the ICM and many other astrophysical systems, heat transport operates in a highly magnetized regime in the sense that the ratio between the mean free path for energy exchange between electrons, $\lambda _{\textrm{mfp}}^{\textrm{e,e}}$ (Spitzer 1956), and the electron gyroradius $r_{e}$ is many orders of magnitude larger than unity. Due to this scale separation, charged particles are tightly bound to magnetic-field lines and heat transport perpendicular to the field is strongly suppressed relative to the parallel transport. This implies that the efficiency of heat transport will be highly anisotropic, having a strong dependence on the orientation of the magnetic field with respect to the temperature gradient in the system and/or on the entanglement of the field lines.

Besides its dependence on the large-scale field configuration, anisotropic heat transport may significantly affect the evolution of the large-scale field itself through buoyancy instabilities. For instance, the magnetothermal instability (MTI) can arise when the plasma temperature gradient is parallel to gravity (Balbus 2000), giving rise to significantly entangled magnetic-field configurations (e.g., McCourt et al. 2011). The required conditions for the MTI are expected to occur in the outskirts of galaxy clusters (Kempf et al. 2023), and it can also be present in hot dilute accretion flows (Bu et al. 2011). Similarly relevant for the creation of entangled fields is the heat-flux-driven buoyancy instability (HBI; Quataert 2008). The HBI acts when the temperature gradient is anti-parallel to gravity, which is the case in the center of galaxy clusters. This instability tends to rearrange magnetic fields to a tangential configuration, suppressing heat transport between different radii in clusters (Parrish et al. 2009; Bogdanović et al. 2009).

Furthermore, many astrophysical environments are in the weakly collisional regime, meaning the ratio between $\lambda _{\textrm{mfp}}^{\textrm{e,e}}$ and the macroscopic temperature gradient length-scale, $L_{T_{e}}$ can be ∼1 or even much larger than unity, as seen in Fig. 1. In these weakly collisional, magnetized plasmas, anisotropic viscous stresses and wave-particle interactions modify the MTI and HBI appreciably (Kunz 2011; Kunz et al. 2012; Xu and Kunz 2016). Furthermore, kinetic instabilities are expected to play a crucial role in the regulation of heat transport. Indeed, the heat transport itself implies a distortion of the distribution function, which can provide free energy for the excitation of micro-scale kinetic instabilities (e.g., Bott et al. 2024). For large plasma beta, small amounts of transport are enough to excite these instabilities, which can scatter and/or trap particles, thereby limiting the transport.

In this context, the oblique heat-flux-driven whistler instability has attracted much attention in recent years. This instability was first investigated theoretically by Pistinner and Eichler (1998), where a heat flux limit $\propto \beta _{e}^{-1}$ was found. Later, this result was confirmed numerically using 2D particle-in-cell (PIC) simulations by Roberg-Clark et al. (2018b), Komarov et al. (2018), Yerger et al. (2025). This instability is expected to limit parallel heat transport below Spitzer expectations for systems with sufficiently large plasma beta, as is the case of the ICM and several other astrophysical systems (see Fig. 1). This implies that kinetic, electron-scale instabilities are expected to play a key role in regulating the global properties and evolution of weakly collisional astrophysical plasmas, determining their thermodynamic evolution as well as their large-scale magnetic-field configurations, e.g., via buoyancy instabilities (Perrone et al. 2024a). This makes heat transport in weakly collisional astrophysical systems an extreme multi-scale problem, where kinetic electron-scale physics strongly affects their large-scale evolutions.

### Laboratory Plasmas

The study of ionised gasses in the laboratory provided the first identification of plasma waves such as the electrostatic Langmuir wave and the ion-acoustic wave, where electron transport is integral to the dynamics (Tonks and Langmuir 1929). Early studies of laboratory plasma generated some classic plasma physics problems. For example, the Spitzer problem arose when studying plasma confinement in stellarator devices (Spitzer and Härm 1953). The problem consists in the fact that steady-state relationship between the current and electric field ultimately requires detailed description of the electron collision behavior due to deformations of the electron distribution function: providing this description proved not to be an easy task, see Sect. 1.1.

The inability of collisions to restore electrons to local thermodynamic equilibrium at high temperatures leads to another famous problem, called “runaway electrons”, where increasingly fast electrons are decreasingly constrained by Coulomb collisions, thereby obtaining high speeds and driving strong electrical currents that often become unstable and generate waves (Dreicer 1959, 1960).

Other fundamental laboratory physics observations which pointed towards the importance of kinetic physics in the transport and evolution of heat flux include the experimental verification of the Landau damping of Langmuir waves (Malmberg and Wharton 1964), the ion-cyclotron and electron-Landau damping of shear Alfvén waves (Vincena et al. 2001), whistler mode to lower-hybrid conversion due to density inhomogeneities (Bamber et al. 1995), and verification of the plasma wave echo (Malmberg et al. 1968).

Laboratory plasmas of particular scientific and societal relevance are fusion plasmas. These plasmas are of particular interest for the non trivial connection between heat transport (the topic of this paper) and flow transport, i.e. the movement of mass and momentum within plasmas. These two aspects are connected due to the fact that temperature gradient instabilities drive significant particle fluxes.

Gas-like collisional heat transport is irrelevant in fusion plasmas; however, collisions of particles on magnetically trapped orbits – or commonly labeled *banana* orbits due to their projected shape – lead to large radial displacements (by a banana width) and can thus produce non-negligible losses. Such transport is referred to as neoclassical flux. In modern fusion devices, however, neoclassical transport is strongly subdominant to anomalous (or turbulent) transport.

Turbulent transport is the consequence of the concurrence of gyroradius-scale electromagnetic fluctuations and background temperature or density gradients, both of which are ubiquitous in fusion plasmas. These gradients constitute a free-energy source that leads to drift-wave instabilities. While fluid models capture linear properties of these mechanisms (Holland et al. 2003), wave-particle resonances are important in these low-collisionality plasmas, and the use of kinetic frameworks is imperative, especially when seeking the quantitative reproduction of experimental fluctuation amplitudes or fluxes (Brizard and Hahm 2007).

Many of the drift-wave instabilities that limit plasma confinement in fusion experiments are electrostatic and thus rely only on the electric potential rather than on magnetic perturbations for their instability mechanism. Important examples in this category of unstable modes are the ion-temperature-gradient (Romanelli 1989) and electron-temperature-gradient modes (Nevins et al. 2006) as well as the trapped-electron mode (Coppi and Rewoldt 1974); the electron-temperature-gradient mode is the only driver of turbulence at the electron gyroradius scale, while the other instabilities arise at the ion gyroradius scale. The electric potential created by the electrostatic instabilities produces (perturbed) ${\mathbf{E}} \times {\mathbf{B}}$ drifts, which lead to electrostatic heat and particle fluxes. In addition, these waves have – often small – magnetic components, whose magnitude scales with $\beta _{\mathrm{e}}$. When electrons follow perturbed field lines, they effectively travel radially through the flow along the field line; this leads to magnetic flutter transport (Rechester and Rosenbluth 2020; Pueschel and Jenko 2010). In order for particles to be effectively displaced in this fashion, flux surfaces must be broken on the scale of the ion gyroradius due to field stochastization, which is ubiquitous in fusion plasmas (Nevins et al. 2011).

Heat and particle transport in fusion plasmas is a multi-scale problem. Setting aside the separation between transport and turbulence scales, electron temperature-gradient-turbulence can impact and be impacted by drift-wave turbulence at the ion gyroscale (Görler and Jenko 2008; Pueschel et al. 2020). The mechanisms of cross-field transport are not exactly known, but in many scenarios the magnetic fluctuations are large, and so it is reasonable to conclude that the lighter, faster electrons are primarily responsible for heat transport. On the contrary, the electrostatic modes tend to produce less disparate fluxes between species, and, in many cases, the ion channel can dominate over the electron channel. Factors such as magnetic geometry and fueling must then be taken into account to determine whether energy can be passed on to the reactant ion species before energy dissipates into the electrons via turbulent mechanisms.

Although collisions are typically of minor importance in fusion plasmas, in weakly collisional scenarios, mode resonances can be affected by collisions and collisionality can in fact enhance mode growth. This is particularly the case for the dissipative trapped-electron mode (Ernst et al. 2004), certain microtearing regimes (Drake and Lee 1977), as well as for resistive drift waves (Mosetto et al. 2013), which can occur near the plasma edge. Outside the confined plasma region, in the so-called scrape-off layer, no flux surfaces exist, and field lines eventually intersect with the wall. There, the question of parallel versus perpendicular transport becomes pertinent (Zito et al. 2021); which of the two is more relevant generally depends on a variety of factors, including the divertor geometry (Ryutov et al. 2012), but at present there is a substantial need for additional research to understand all processes in this region of fusion devices.

Lastly, the practical demands imposed by the need to optimize and control fusion reactors, in conjunction with the high computational expense of nonlinear simulations as well as the scale separation between turbulence and transport, have led to research into a hierarchy of reduced transport models. A common approach is to construct quasilinear models (Staebler et al. 2007; Bourdelle et al. 2015) that replace complex nonlinear interactions with mixing-length arguments, allowing the evaluation of heat and particle fluxes based on a moderate number of linear simulations.

### Earth’s Magnetosphere and Ionosphere

In the Earth’s magnetosphere— and in particularly in the inner magnetosphere— electron dynamics is primarily controlled by the strong dipolar magnetic field. Electrons bounce rapidly along magnetic field lines (with bounce periods ranging from 0.1 to 10 s for energies of 100 keV to 1 keV), and this bouncing establishes velocity distributions that are symmetric in $v_{\parallel}$, producing no net heat flux. However, the magnetosphere is coupled to a natural conducting boundary: the Earth’s ionosphere, which is filled with dense, cold, and collisional plasma.

Wave–particle interactions in the equatorial magnetosphere are the primary driver of $0.1-10\text{ keV}$ electron precipitation into the ionosphere. The dominant wave modes responsible for such scattering and precipitation include whistler-mode waves, electron cyclotron harmonics, and electrostatic turbulence (Zhang et al. 2015; Ni et al. 2016; Vasko et al. 2017; Shen et al. 2021). These precipitating electrons generate a secondary electron population via collisions and, together with the primary precipitation, undergo complex magnetosphere–ionosphere coupling before transferring energy to the ionospheric core plasma (see Khazanov 2011; Khazanov et al. 2014). The energy deposited into the core plasma ultimately returns to the ionosphere in the form of the electron thermal heat flux, which supports elevated electron temperatures at upper ionospheric altitudes.

Thermal conduction is one of the primary processes governing electron energy transport in the ionosphere. This conduction is controlled by collisions with neutrals, ions, and electrons. At high altitudes, inelastic collisions are negligible compared with Coulomb collisions; consequently, the energy transport may be treated as conservative.

An analytical expression for the electron thermal heat flux can be written (see Schunk 1977, and references therein) as

13 $$ q_{e}^{\mathrm{Schunk 1977}}=-\alpha ^{*} \, T_{e}^{5/2} \frac{\partial T_{e}}{\partial R} $$

where $\alpha ^{*}$ is a constant derived from the electron conductivity and R is the radial distance.

The standard approach for estimating the thermal heat flux into the ionosphere, and for evaluating its impact on ionospheric density profiles, involves determining $T_{e}$ profiles from incoherent-scatter radar measurements (e.g., Blelly and Alcayde 1994; Fallen and Watkins 2013) and conducting numerical simulations of ionospheric dynamics with various upper-boundary heat-flux values (e.g., Bekerat et al. 2007; Khazanov et al. 2020). Model plasma densities can then be validated using measurements from low-altitude spacecraft such as the Swarm (Friis-Christensen et al. 2008) or Defense Meteorological Satellite Program (DMSP) missions (Kilcommons et al. 2017).

**Fig. 3:**
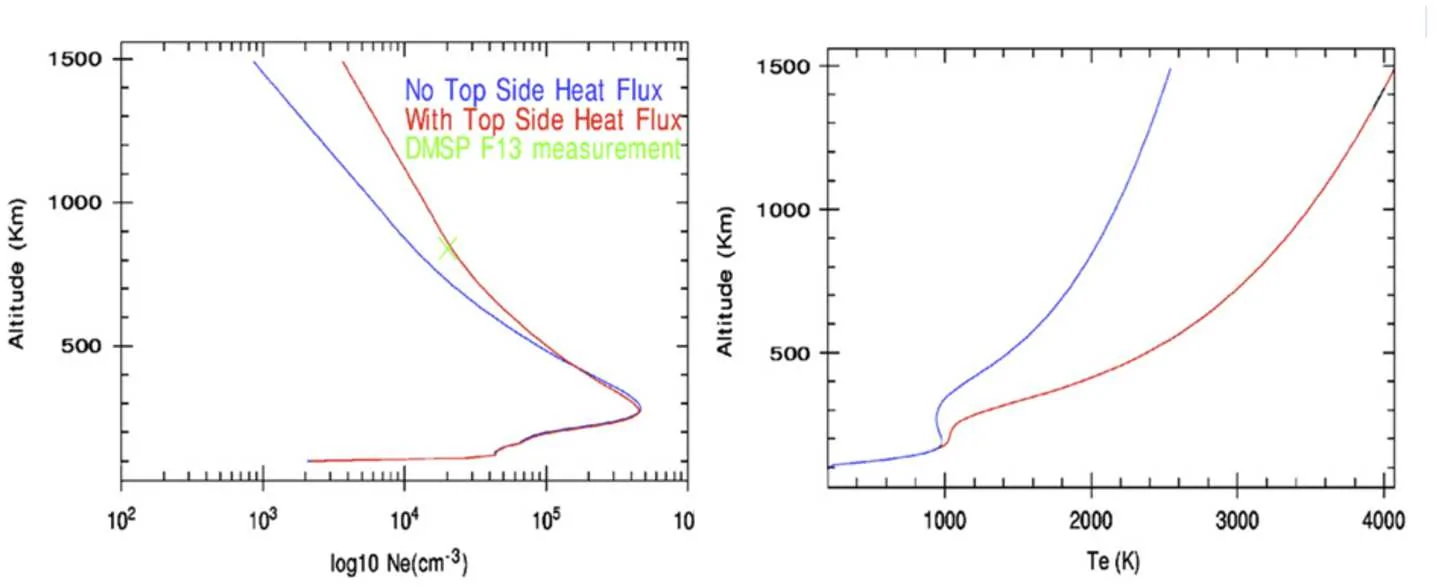
Snapshots of electron temperature, $T_{e}$, and plasma density, $N_{e}$, versus altitude from an ionospheric simulation corresponding to a DMSP satellite pass over the northern polar region. Blue curves were calculated with no topside heat flux; red curves were calculated with the appropriate topside heat flux; and the green cross shows the DMSP measurement. Figure adapted from Bekerat et al. (2007)

Figure 3 shows an example of an ionospheric simulation that includes a realistic heat flux. The upper-boundary heat flux produces substantial variations in electron temperature and density, $T_{e}$ and $n_{e}$, with altitude. The conducted heat raises $T_{e}$ at all altitudes down to roughly 200 km; below this level, local heating and cooling processes dominate. The increased electron temperatures decrease plasma densities at lower altitudes and increase densities at higher altitudes. The measured plasma densities are nearly a factor of two larger than those from simulations with no upper-boundary heat flux. When the appropriate heat-flux value is used, agreement between the simulated and measured plasma densities improves significantly (for details, see Bekerat et al. 2007). Overall, these results demonstrate the importance of electron heat flux in ionospheric heating (see also discussion by Ridley et al. 2006).

Heat-flux transport and ionospheric heating are analogous to the heating of the solar corona due to energetic electron precipitation from energy-release regions (Aschwanden et al. 2017; Warmuth and Mann 2020), but differ markedly from heat-flux transport in the collisionless solar wind, where the electron conductivity cannot be described by $\kappa _{e}=\alpha ^{*} T_{e}^{5/2}$, see Sect. 1.4 and Sect. 2.1.

## Transport Theory: Collisional, Collisionless, and in Between

**Fig. 4:**
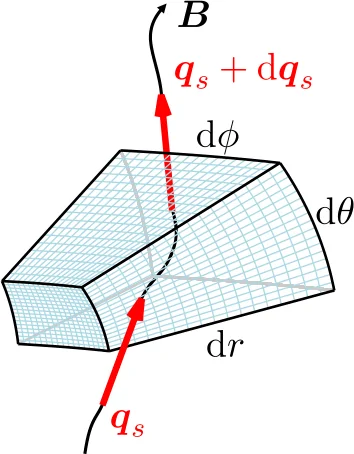
Illustration of heating or cooling through a non-zero divergence of the heat flux in a volume element in spherical coordinates. The heat flux $\boldsymbol{q}_{s}$ enters a volume element along the magnetic field $\boldsymbol{B}$. If processes inside the volume element change the heat flux by an amount $\mathrm {d}\boldsymbol{q}_{s}$, then heat flux deposition or removal can lead to a change in thermal energy in the volume element

Most of transport theory is concerned with closure at the level of the heat flux $\boldsymbol{q}_{s}$, and so we focus our attention there. The regulation of $\boldsymbol{q}_{s}$ impacts the thermal energy budget of a given plasma volume. The energy equation (i.e., a variety of the second moment of the Vlasov equation) describes the variation in time of the second moment of the velocity distribution function, namely the scalar-isotropic pressure $p_{s}$. When neglecting the pressure strain terms and the collisional term, this yields

14 $$ 3\left (\frac{\partial }{\partial t}+\boldsymbol{u}_{s}\cdot \nabla \right ) p_{s} + 5p_{s}\nabla \cdot \boldsymbol{u}_{s}+\nabla \cdot \boldsymbol{q}_{s} =0, $$

where the drift velocity $\boldsymbol{u}_{s}$ is related to the first velocity moment of the velocity distribution function, the heat flux tensor $\boldsymbol{q}_{s}$ is the centered third moment, and we use scalar-isotropic pressure $p_{s}$ in place of the pressure tensor, i.e. the centered second moment of the velocity distribution function. Equation (14) highlights that the heat flux contributes to a change in internal energy density if and only if $\nabla \cdot \boldsymbol{q}_{s}\ne 0$. This means that, although a plasma population like the electrons in the solar wind carry a large amount of heat flux, this kinetic property of the population only has an effect on the population’s thermal behaviour if the heat flux vector has sources or sinks. Figure 4 illustrates the effect of sources or sinks in $\boldsymbol{q}_{s}$. The figure shows an infinitesimal volume element in spherical coordinate space. The black curve represents a magnetic field line that passes through the volume element. We assume that a particle population carries the heat flux $\boldsymbol{q}_{s}$ into the volume element along the magnetic field. If processes inside the volume element change the heat flux, then $\nabla \cdot \boldsymbol{q}_{s}\neq 0$ and heat flux deposition or removal contributes to the change of the thermal energy of the particles in the volume element.

We can extend this consideration from infinitesimal volume elements like the one shown in Fig. 4 to larger compact volumes with piecewise smooth boundaries by integration. By integrating Eq. (14) over such a volume V, we obtain

15 $$ 3\iiint _{V} \left [\frac{\partial p_{s}}{\partial t}+\nabla \cdot \left (p_{s}\boldsymbol{u}_{s}\right )\right ] \,\mathrm {d}V +2 \iiint _{V} p_{s}\nabla \cdot \boldsymbol{u}_{s}\,\mathrm {d}V+ \iiint _{V} \nabla \cdot \boldsymbol{q}_{s} \,\mathrm {d}V =0. $$

With Gauss’ divergence theorem, we then find

16 $$ \frac{\partial \mathcal {E}_{s}}{\partial t}=-\oiint _{S} p_{s} \boldsymbol{u}_{s}\, \cdot \mathrm {d}\boldsymbol{s}-\frac{2}{3} \iiint _{V} p_{s}\nabla \cdot \boldsymbol{u}_{s}\,\mathrm {d}V- \frac{1}{3}\oiint _{S} \boldsymbol{q}_{s} \cdot \mathrm {d} \boldsymbol{s}, $$

where S=∂V is the closed boundary of volume V and the vector $\mathrm {d}\boldsymbol{s}$ is the outward pointing normal vector of S. We define the total thermal energy in volume V as

17 $$ \mathcal {E}_{s}=\iiint _{V} p_{s}\,\mathrm {d} V. $$

The first term on the right-hand side of Eq. (16) represents the change of thermal energy of species s due to a net flux of thermal energy density that is advected into or out of the volume V. The second term represents the integrated effect of converging or diverging flows (i.e., compressive effects) that change the number of particles and thus their thermal energy inside the volume V. The third term represents the change of thermal energy due to net flux of heat into or out of the volume. This term illustrates that heat fluxes only contribute to changes in $\mathcal {E}_{s}$ in a given compact volume V if the heat flux integrated over the surface of the volume V is non-zero.

Following on from these considerations, the regulation of heat flux is considered relevant for the overall thermal evolution of the plasma if and only if there are processes in a given plasma volume that modify the net flux of heat into or out of the volume. Both collisional and collisionless processes are able to modify the shape of the velocity distribution in such a way that its third moment changes, resulting in $\nabla \cdot \boldsymbol{q}_{s}\ne 0$. Higher-order moment closures are a mathematical way to quantify these effects in fluid models.

**Fig. 5:**
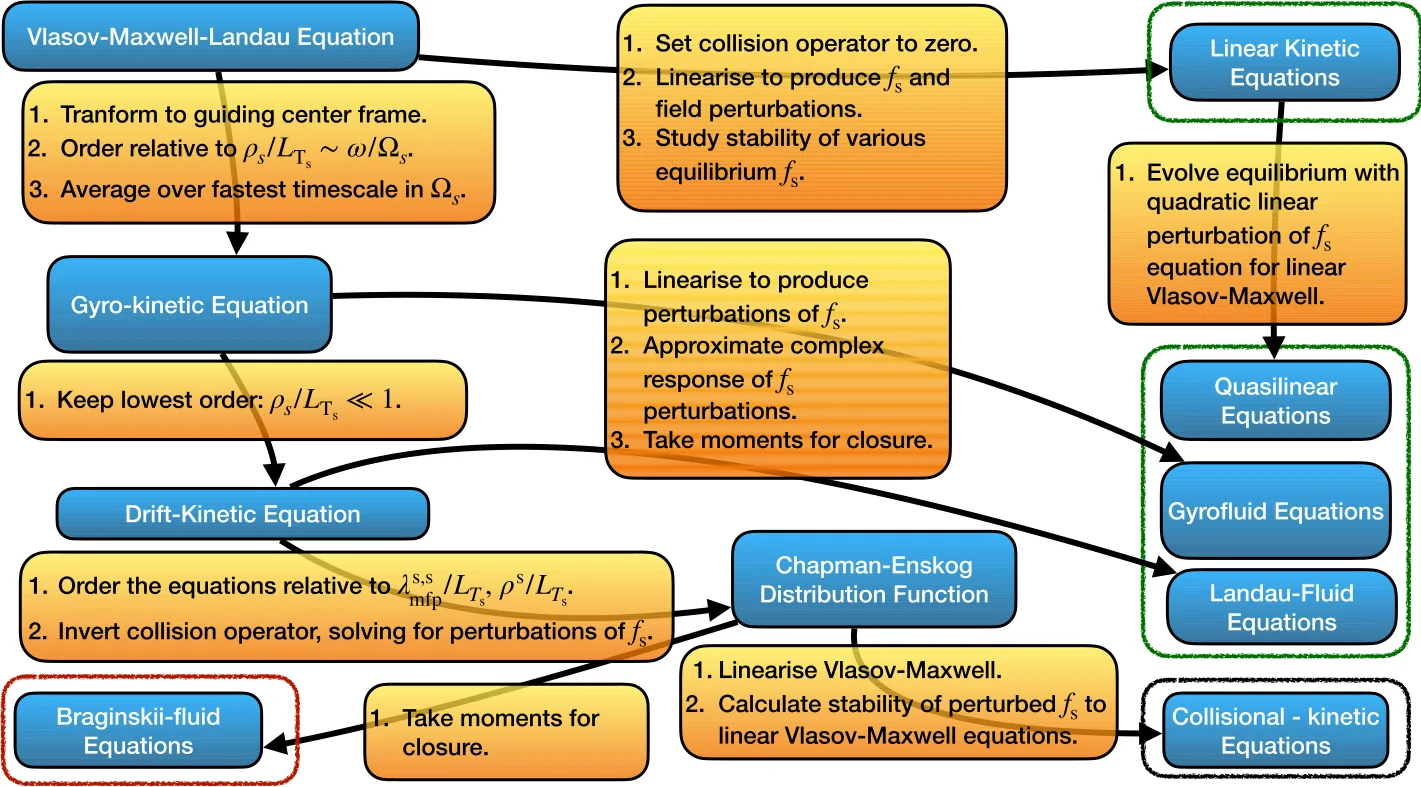
A flow chart providing a basic outline of the assumptions and derivations which lead to key results in transport theory. The blue filled boxes are important sets of equations. The yellow filled boxes are the relevant assumptions invoked to derive the equations. In the text we describe the avenues of the derivations, and point to specific texts that provide exemplary details. The non-filled maroon box contains results in collisional theory (Sect. 3.1), the non-filled green boxes are results in collisionless theory (Sect. 3.2), and the non-filled black box highlights theory that bridges the two regimes (Sect. 3.3)

Collisional and collisionless higher-order moment closures have been thoroughly derived and reviewed in classic literature. To provide more detail, without repeating derivations here, we point to a set of lecture notes, reviews, and textbooks that detail the derivations we discuss in this article. Figure 5 presents a flow chart of the equations and a description of the derivations.

The derivation of the “Vlasov-Maxwell-Landau” equation is well-covered in many textbooks, but in depth details on the collision operator (i.e. the reason for including Landau and Boltzmann in the name) can be found in Kunz (2003) and Hunana (2025). The procedure connecting “Vlasov-Maxwell-Landau” to the “Gyro-kinetic Equation” can be found in the review by Brizard and Hahm (2007). The “Drift-kinetic Equation” predates the “Gyro-kinetic Equation”, and can be found in Hazeltine et al. (1975), Kulsrud (1983), and an enlightening derivation that connects the two equations can be found in Hunana et al. (2019b).

The derivation that connects the “Drift-kinetic Equation” to the “Chapman-Enskog Distribution Function” can be found in Braginskii (1965), Kunz (2003), Hunana et al. (2019a), Bott et al. (2024). The derivation connecting “Chapman-Enskog Distribution Function” to “Braginskii-fluid Equations” is found in Braginskii (1965), Kunz (2003), Hunana et al. (2019a). To connect “Chapman-Enskog Distribution Function” to “Collisional - kinetic Equations”, an excellent demonstration can be found in Bott et al. (2024), and for a related but slightly different avenue see Pistinner and Eichler (1998).

The derivation connecting the “Gyro-kinetic Equation” to the “Gyrofluid Equations” can be found in Dorland and Hammett (1993) and Beer and Hammett (1996). We do not explicitly discuss the gyrofluid equations in this article, but one can see that the procedure is similar to the connection between the “Drift-kinetic” and the “Landau-Fluid Equations”. The essential difference is that gyrofluid equations retain finite-Larmour-radius effects. The derivation connecting the “Drift-kinetic Equation” to the “Landau-Fluid Equations” can be found in Hammett and Perkins (1990), Hunana et al. (2019a).

The connection between “Vlasov-Maxwell-Landau” and the “Linear Kinetic Equations” can be found in Gary (1993). An essential review on these results, with an emphasis on the role electron microinstabilities, is Verscharen et al. (2022a). Finally, the connection between “Linear Kinetic Equations” and the “Quasilinear Equations” can be found in Yoon (2017), with notable work on the topic by Astfalk and Jenko (2018).

### Collisional Transport Theory

As shown in Fig. 5, the essential idea is to perform an expansion in $\lambda _{\mathrm{mfp}}^{\mathrm{s,s}}/L_{T_{\mathrm{s}}}, \; \rho ^{ \mathrm{s}}/L_{T_{\mathrm{s}}}$ to obtain a closed form of the electron and ion distribution functions. Moments of the resulting distribution functions provide the heat fluxes, friction forces, viscosity tensor, and thermal energy transfer which close the equations. The ordering is called the Chapman–Enskog expansion, see Kunz (2003) for a thorough explanation.

Considerable understanding of how electrons mediate transport in collisional plasmas can be obtained by comparing the relative time scales of the four collision frequencies that arise in an electron and single ion plasma. Collisions, even with assumptions of the Bogoliubov–Born–Green–Kirkwood–Yvon hierarchy and those that lead to Landau’s collision operator (cf. Kunz 2003), are complicated. We redirect to any classic text for collisional transport theory (see Sect. 1) and for a recent investigation of the Boltzmann collision operator we refer the reader to Hunana (2025). Most collision time scales are computed for simple arrangements, i.e. a quasi-Maxwellian plasma is interacting with a test-particle or drifting Maxwellian. For recent work on numerical simulations of proton-electron plasmas with sophisticated collision operators see Pezzi et al. (2016, 2019a,b) where fine-structure in the VDF are shown to relax towards marginal stability over shorter time scales with respect to the dissipation of global (i.e., larger scale) non Maxwellian features.

For our purposes, the collision frequencies are approximated to

18 $$\begin{aligned} &\nu _{\mathrm{e,e}} \approx 3.6 \times 10^{-6} \, \mathrm{ln} | \Lambda _{\mathrm{e,e}} | \frac{n_{\mathrm{e}}}{\tilde{T}_{\mathrm{e}}^{3/2}}, \end{aligned}$$

19 $$\begin{aligned} &\nu _{\mathrm{p,p}} \approx 5.8 \times 10^{-8} \, \mathrm{ln} | \Lambda _{\mathrm{p,p}} | \frac{n_{\mathrm{p}}}{\tilde{T}_{\mathrm{p}}^{3/2}}. \end{aligned}$$

The Coulomb logarithm $\mathrm{ln} |\Lambda _{\mathrm{s,s}} |$, with s the plasma species, appears in Huba (1998) where the $\tilde{T}_{s}$ must be in electron volts. The collision frequencies above are valid for rates considering equilibration and momentum transfer. The notation $\nu _{\mathrm{s,s'}}$ indicates the evolution of s due to collisions with $s^{\prime }$. The four frequencies in an electron-proton plasma scale as

20 $$\begin{aligned} \nu _{\mathrm{e,e}}, \nu _{\mathrm{e,p}} \; : \; \nu _{\mathrm{p,p}} \; : \; \nu _{\mathrm{p,e}} = 1 \; : \bigg( \frac{m_{\mathrm{e}}}{m_{\mathrm{p}}} \bigg)^{1/2} \; : \; \frac{m_{\mathrm{e}}}{m_{\mathrm{p}}} \end{aligned}$$

so that electron self-collisions and electrons-proton collisions dominate the hierarchy of time scales. Protons are primarily modified by self-collisions. The much larger mass of the protons modifies the less massive electrons considerably, reflected in the time scale hierarchy, and the modification of protons by collisions with electrons can often be neglected.

For a general understanding of collision rates in the solar wind at 1 au, in Table 2, we report collision rates between particle species s and $s^{\prime }$ following the notation from Wilson et al. (2018), with the updated data from Wilson et al. (2023).

**Table 2:** Reference parameters used to calculated the quantities depicted in Fig. 1, Fig. 2, and Fig. 7, for the different environments. The solar wind and solar corona do not appear in the table, but appear directly in the figures and are explained in the text

Collision rates measured in the solar wind				
Collision rate	Lower-upper quartiles	Mean	Median	Unit
$\nu _{\mathrm{e,e}}$	0.21–0.62	0.48	0.34	collisions/day
$\nu _{\mathrm{p,p}}$	0.01–0.09	0.07	0.03	collisions/day
$\nu _{{\alpha ,\alpha}}$	0.001–0.01	0.01	0.005	collisions/day
$\nu _{\mathrm{e,p}}$	0.19–0.58	0.45	0.31	collisions/day
$\nu _{\mathrm{e},\alpha}$	0.03–0.08	0.07	0.05	collisions/day
$\nu _{\mathrm{p},\alpha}$	0.002–0.008	0.007	0.004	collisions/day

The α denotes He^2+^ particles, with relative proton densities ($n_{\mathrm{p}}/n_{\alpha}$) spanning from 0.01 to 0.20 with an average of 0.05.

Several of these parameters from a statistical dataset have been provided in more detail in other works e.g., see Wilson et al. (2018, 2021b,a, 2023).

As discussed earlier, the primary task of a collisional theory is to employ the Chapman–Enskog expansion to obtain closure. Typically, the equations are closed at the level of the heat flux, where the electron heat flux dominate when $T_{\mathrm{e}} \approx T_{\mathrm{p}}$. Here we write the electron heat flux

21 $$\begin{aligned} {\mathbf{q}}^{\mathrm{e}} &= -\kappa _{\parallel , T}^{\mathrm{e}} \hat{\mathbf{b}} ( \hat{\mathbf{b}} \cdot {\mathbf{\nabla}} T_{\mathrm{e}}) - \kappa _{\perp , T}^{\mathrm{e}} \big[ {\mathbf{\nabla}} T_{\mathrm{e}} - \hat{\mathbf{b}} ( \hat{\mathbf{b}} \cdot {\mathbf{\nabla}} T_{\mathrm{e}}) \big] + \kappa _{\times , T}^{\mathrm{e}} \, \hat{\mathbf{b}} \times {\mathbf{\nabla}} T_{ \mathrm{e}} \\ & + \kappa _{\parallel ,u}^{\mathrm{e}} \, \hat{\mathbf{b}} \big[ \hat{\mathbf{b}} \cdot ({\mathbf{u}}^{\mathrm{e}} - {\mathbf{u}}^{\mathrm{p}}) \big] + \kappa _{\perp , u}^{\mathrm{e}} \, \big\{ ({\mathbf{u}}^{\mathrm{e}} - { \mathbf{u}}^{\mathrm{p}}) - \hat{\mathbf{b}} [ \hat{\mathbf{b}} \cdot ({\mathbf{u}}^{ \mathrm{e}} - {\mathbf{u}}^{\mathrm{p}}) ]\big\} + \kappa _{\times , u}^{ \mathrm{e}} \hat{\mathbf{b}} \times \big({\mathbf{u}}^{\mathrm{e}} - {\mathbf{u}}^{ \mathrm{p}} \big), \end{aligned}$$

where

22 $$\begin{aligned} &\kappa _{\parallel , T}^{\mathrm{e}} = \hat{\kappa}_{\parallel , T}^{ \mathrm{e}} \frac{k_{\mathrm{B}} \, p_{\mathrm{e}}}{\nu _{\mathrm{e,e}} m_{\mathrm{e}}}, \; \kappa _{\perp , T}^{\mathrm{e}} = \hat{\kappa}_{\perp , T}^{ \mathrm{e}} \frac{k_{\mathrm{B}} \, p_{\mathrm{e}}}{ \nu _{\mathrm{e,e}} m_{\mathrm{e}}}, \; \kappa _{\times}^{\mathrm{e}} = \hat{\kappa}_{\times , T}^{ \mathrm{e}} \frac{k_{\mathrm{B}} \, p_{\mathrm{e}}}{\nu _{\mathrm{e,e}} m_{\mathrm{e}}} \\ &\kappa _{\parallel , u}^{\mathrm{e}} = \hat{\kappa}_{\parallel , u}^{ \mathrm{e}} p_{\mathrm{e}} , \; \kappa _{\perp , u}^{\mathrm{e}} = \hat{\kappa}_{\perp , u}^{\mathrm{e}} p_{\mathrm{e}}, \; \kappa _{ \times}^{\mathrm{e}} = \hat{\kappa}_{\times , u}^{\mathrm{e}} p_{ \mathrm{e}}. \end{aligned}$$

and the momentum transfer rates are

23 $$\begin{aligned} {\mathbf{R}}_{\mathrm{e}} &= - \tau _{\parallel , u}^{\mathrm{e}} \hat{\mathbf{b}} \big[ \hat{\mathbf{b}} \cdot ({\mathbf{u}}^{\mathrm{p}} - {\mathbf{u}}^{ \mathrm{e}} ) \big] - \tau _{\perp , u}^{\mathrm{e}} \big\{ ( {\mathbf{u}}^{ \mathrm{p}} - {\mathbf{u}}^{\mathrm{e}}) - \hat{\mathbf{b}} [ \hat{\mathbf{b}} \cdot ({\mathbf{u}}^{\mathrm{p}} - {\mathbf{u}}^{\mathrm{e}}) ] \big\} - \tau _{ \times , u}^{\mathrm{e}} \hat{\mathbf{b}} \times \big({\mathbf{u}}^{\mathrm{p}} - {\mathbf{u}}^{\mathrm{e}} \big) \\ & \hspace{0.75cm} - \tau _{\parallel , T}^{\mathrm{e}} \hat{\mathbf{b}} ( \hat{\mathbf{b}} \cdot { \mathbf{\nabla}} T_{\mathrm{e}} ) - \tau _{\perp , T}^{\mathrm{e}} \big[ { \mathbf{\nabla}} T_{\mathrm{e}} - \hat{\mathbf{b}} (\hat{\mathbf{b}} \cdot {\mathbf{\nabla}} T_{\mathrm{e}}) \big] - \tau _{\times , T}^{\mathrm{e}} \hat{\mathbf{b}} \times {\mathbf{\nabla}} T_{\mathrm{e}} , \\ {\mathbf{R}}_{\mathrm{p}} &= - {\mathbf{R}}_{\mathrm{e}}. \end{aligned}$$

The hatted coefficients are named with subscripts, where ∥ – parallel transport, ⊥ – perpendicular transport, × – non-gyrotropic transport. This particular form is derived in Hunana et al. (2022) for a proton-electron plasma. It is typically derived in a limit of what we call

24 $$\begin{aligned} \mathrm{Braginskii} \; \mathrm{ratio} = \frac{\lambda _{\mathrm{mfp}}^{\mathrm{e,e}}}{\rho _{\mathrm{e}}} = \frac{|\Omega _{\mathrm{e}}|}{\nu _{\mathrm{e,e}}}. \end{aligned}$$

**Fig. 6:**
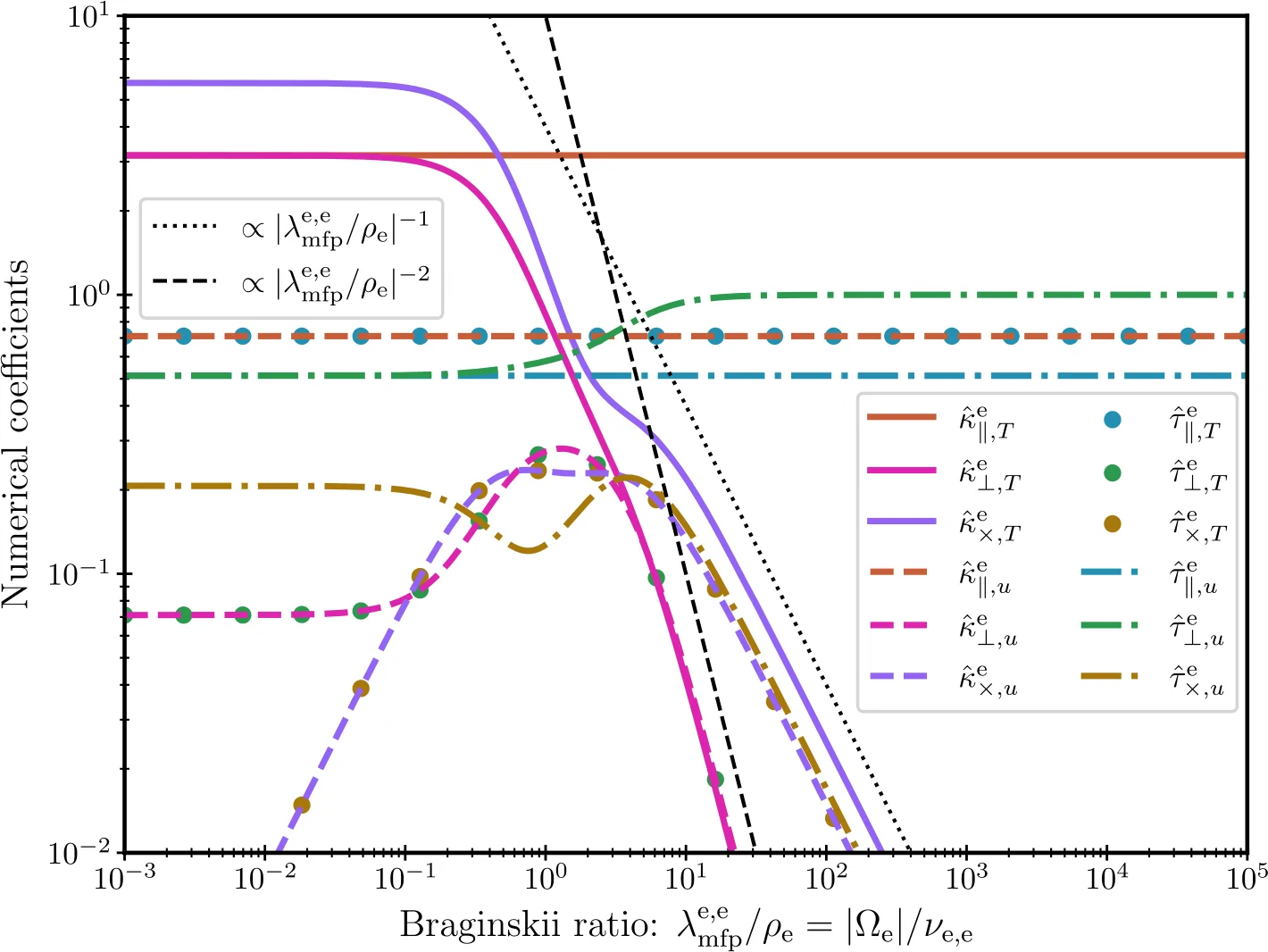
The dependence on the Braginskii ratio (Eq. (24)) of the kinetic coefficients in the electron heat flux formula (Eq. (21)) and in the momentum transfer rates formula (Eq. (23)). For reference, two black lines (dotted and dashed) are shown, as they govern the power law decrease of the perpendicular (⊥) and non-gyrotropic (×) terms as $\lambda _{\mathrm{mfp}}^{\mathrm{e,e}} / \rho _{\mathrm{e}}$ increases. In Fig. 7 we plot the plasma environments from Table 1 for reference

Here, we rewrite them to be unitless

25 $$\begin{aligned} & \tau _{\parallel , u}^{\mathrm{e}} = \hat{\tau}_{\parallel , u}^{ \mathrm{e}} \, m_{\mathrm{e}} n_{\mathrm{e}} \nu _{\mathrm{e,e}}, \; \tau _{\perp , u}^{\mathrm{e}} = \hat{\tau}_{\perp , u}^{\mathrm{e}} \, m_{\mathrm{e}} n_{\mathrm{e}} \nu _{\mathrm{e,e}}, \; \tau _{ \times , u}^{\mathrm{e}} = \hat{\tau}_{\times , u}^{\mathrm{e}} \, m_{ \mathrm{e}} n_{\mathrm{e}} \nu _{\mathrm{e,e}} \\ &\tau _{\parallel , T}^{\mathrm{e}} = \hat{\tau}_{\parallel , T}^{ \mathrm{e}} \, k_{\mathrm{B}} n_{\mathrm{e}} , \; \tau _{\perp , T}^{ \mathrm{e}} = \hat{\tau}_{\perp , T}^{\mathrm{e}} \, k_{\mathrm{B}} n_{ \mathrm{e}} , \; \tau _{\times , T}^{\mathrm{e}} = \hat{\tau}_{ \times , T}^{\mathrm{e}} \,k_{\mathrm{B}} n_{\mathrm{e}}, \end{aligned}$$

and plot them in Fig. 6. We do not discuss the viscosity tensor because it is primarily governed by the ions due to their larger momentum.

From Fig. 6, it is clear that $\lambda _{\mathrm{mfp}}^{\mathrm{e,e}} / \rho _{\mathrm{e}} \gg 1$ perpendicular transport is negligible. Recalling the collisional nature of the theory, it can be understood that very few collisions occur within maybe gyro-periods. In Fig. 6, the coefficients that are marked as dots and dashed lines are equal; this is a consequence of the Onsager symmetry (Onsager 1931a,b). As described by Braginskii (1965), it is a consequences of entropy production in nonequilibrium system.

The hatted coefficients (Eqs. (22) and (25)) appear in Hunana et al. (2022) in various levels of approximation. We have chosen the simplest one which require Eqs. 43, 44, 56-60 from Hunana et al. (2022). We have only considered a proton-electron plasma, but these coefficients do depend on the charge number. There are only a few cases where there is strong dependence on the charge number (cf. Hunana et al. 2022). Each of these coefficients are polynomials of $\lambda _{\mathrm{mfp}}^{\mathrm{e,e}} / \rho _{\mathrm{e}}$. The polynomial form and numerical coefficients are governed by order of moment closure and the form of the collision operator.

**Fig. 7:**
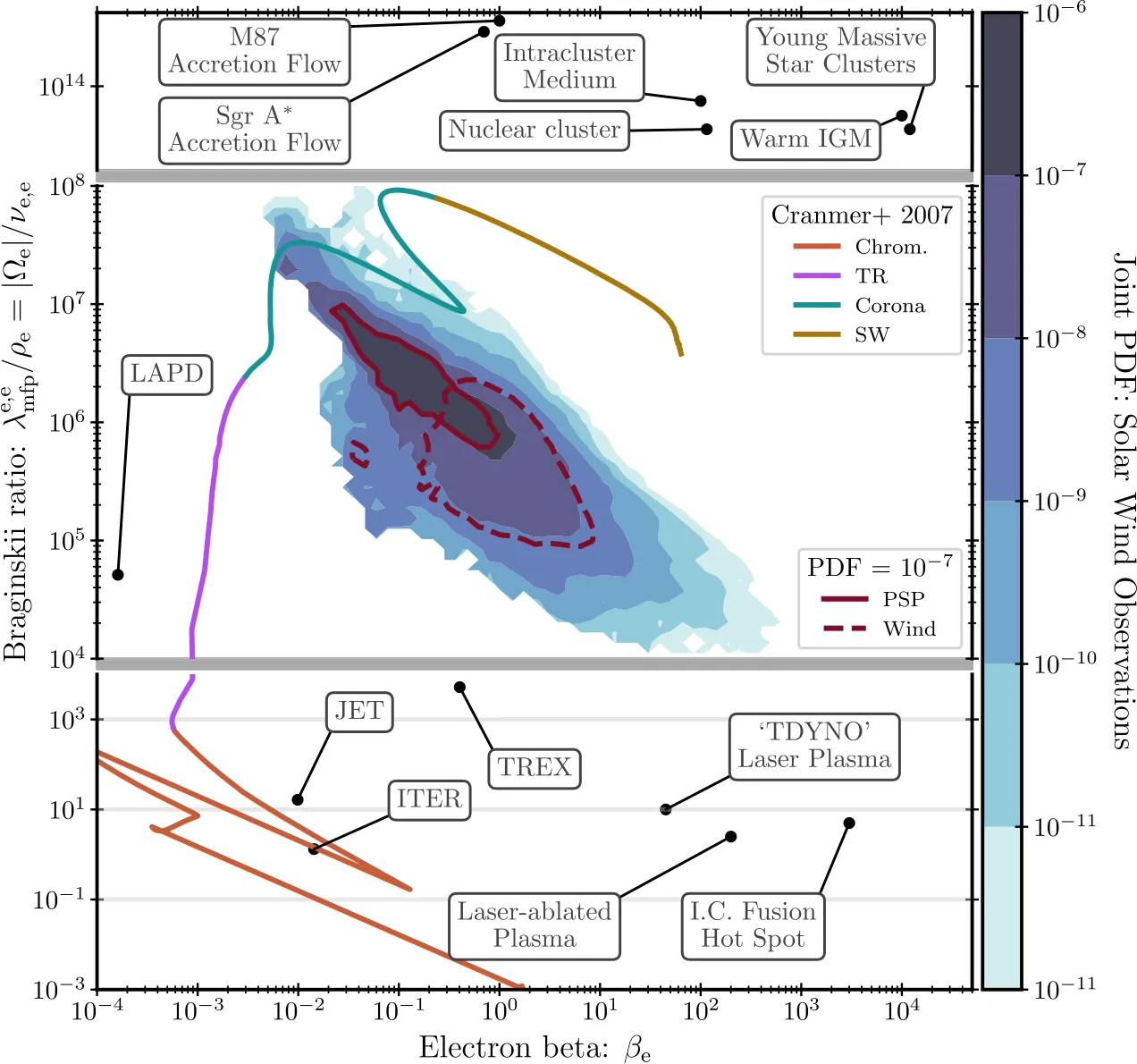
An overview of $\lambda _{\mathrm{mfp}}^{\mathrm{e,e}} / \rho _{\mathrm{e}}$ and the electron beta $\beta _{\mathrm{e}} = 8 \pi n_{\mathrm{e}} k_{\mathrm{B}} T_{ \mathrm{e}} / B_{0}^{2}$ for a set of observations and simulations. The annotated boxes with pointers are observations of laboratory and astrophysical parameters. Citations and values for the observations appear in Table 1. The three horizontal grey lines in the bottom most part of the figure are characteristic values of the Braginskii ratio from, as can be seen in Fig. 6. The joint PDF of solar wind observations in shades of blue is the combined dataset of Parker Solar Probe and Wind (see text for details on the observations). The red contours are the PDF = $10^{-7}$ contour for the individual distributions. The legend titled “Cranmer+ 2007” is a simulation of the solar atmosphere and solar wind by Cranmer et al. (2007) (see the text for details on the simulation). The abbreviated labels are “Chrom.” for chromosphere, “TR” for transition region, and “SW” for solar wind

In Fig. 7 we display the plasma environments from Table 1, the solar wind observations, and results from Cranmer et al. (2007) reflecting the solar atmosphere. The three horizontal grey lines in the bottom most part of the figure are characteristic values of the Braginskii ratio from, as can be seen in Fig. 6. Below $\lambda _{\mathrm{mfp}}^{\mathrm{e,e}} / \rho _{\mathrm{e}} = 10^{-1}$ perpendicular and parallel transport are significant. Above $\lambda _{\mathrm{mfp}}^{\mathrm{e,e}} / \rho _{\mathrm{e}} = 10^{3}$, only parallel transport is significant.

As noted in Fig. 6, the Braginskii coefficient ($\lambda _{\mathrm{mfp}}^{\mathrm{e,e}} / \rho _{\mathrm{e}}$) is a free parameter in the model. This ratio has important implications on the anisotropy of momentum and heat transport. In Fig. 7 we report values for the environments of interest from Table 1. The solar wind is shown as a combined distribution function in blue filled contours, where the PDF = $10^{-7}$ contour for individual spacecraft are shown as red lines (see the legend). We use the simulation results from Cranmer et al. (2007) to show estimates for the solar atmosphere, where both regimes are relevant. In general, astrophysical environments are highly anisotropy, favouring parallel transport, given the small gyroradii in these systems (see Fig. 7). In laboratory systems, perpendicular transport is relevant.

### Regulation of the Heat Flux by Collisionless Processes

Collisionless processes can modify the third moment and thus the heat flux $\boldsymbol{q}_{\mathrm{s}}$ of a given particle species s. In this section we give an overview of different frameworks that can be used to describe heat flux modification by collisionless processes: the Landau fluid approach (Sect. 3.2.1), where one attempts to match the asymptotic limits of the kinetic response function in specific processes (e.g., Landau damping) with a polynomial approximation with appropriately selected coefficients; a closed representation of the value of heat flux at instability threshold (see Sect. 3.2.2), written in terms of coefficients whose values are calculated for different heat-flux-regulating instabilities; and, finally, quasi-linear theory (see Sect. 3.2.3), which provides information on the saturated state of a system.

#### Landau Fluid Closures

The collisionless fluid-like closure was first proposed by Hammett and Perkins (1990) and the resulting set of equations are called the Landau fluid equations. They are fluid-like in the sense that the moment chain is closed, importantly, by approximating linear perturbations of a collisionless kinetic equation, and so they model Landau damping. In an illustrative form, the drift-kinetic equation is considered

26 $$\begin{aligned} &\frac{\partial}{\partial t} f_{s} + \big( w^{s}_{\parallel} \hat{\mathbf{b}} + {\mathbf{u}}^{s} \big) \cdot {\mathbf{\nabla}} f_{s} + \bigg( \frac{\mathrm{d} w^{s}_{\perp} }{\mathrm{d} t} \frac{\partial }{\partial w^{s}_{\perp}} + \frac{\mathrm{d} w^{s}_{\parallel} }{\mathrm{d} t} \frac{\partial }{\partial w^{s}_{\parallel}} \bigg) f_{s} = 0 \end{aligned}$$

27 $$\begin{aligned} &\frac{\mathrm{d} w^{s}_{\perp} }{\mathrm{d} t} = \frac{w^{s}_{\perp}}{2 |{\mathbf{b}}|} \frac{\mathrm{d} |{\mathbf{b}}| }{\mathrm{d} t} = 0 \end{aligned}$$

28 $$\begin{aligned} & \frac{\mathrm{d} w^{s}_{\parallel} }{\mathrm{d} t} = \frac{q_{s}}{m_{s}} E_{\parallel} - \frac{\mu _{s}}{m_{s}} \hat{\mathbf{b}} \cdot {\mathbf{\nabla}} |{\mathbf{b}}| - \hat{\mathbf{b}} \cdot \bigg[ \frac{\partial}{\partial t} + \bigg( w_{\parallel}^{s} \hat{\mathbf{b}} + { \mathbf{u}}^{s} \bigg) \cdot {\mathbf{\nabla}} \bigg] {\mathbf{u}}^{s}. \end{aligned}$$

where $f_{\mathrm{s}} = f_{\mathrm{s}}(t, {\mathbf{x}}, w_{\parallel}, w_{\perp})$ is the gyrotropic distribution function. Equation (27) enforces magnetic moment $\mu _{s} = m_{s} |w_{\perp}|^{2} / 2 |{\mathbf{b}}|$ conservation. In Eq. (28), the first term leads to Landau damping, the second term leads to transit-time damping (Barnes damping), and the final term is conserves inertia of the system in the drifting frame ${\mathbf{v}} + {\mathbf{u}}^{s}$.

A Landau fluid closure is obtained by perturbing Eq. (26), taking moments for closure and approximating the Landau integral. This is typically done with Padé series. For extensive and instructive review of this method see Hunana et al. (2019a). This procedure, when perturbed about a bi-Maxwellian background, produces a parallel heat flux

29 $$\begin{aligned} \bar{q}_{s,\parallel} = - i \frac{2}{\sqrt{\pi}} v^{s}_{\mathrm{th}} n_{s} \frac{k_{\parallel}}{|k_{\parallel}|} \bar{T}_{s,\parallel}. \end{aligned}$$

where the bar quantities are the Fourier amplitude of the perturbation and non-bar quantities are the background quantities. This is the closure reported by Hammett and Perkins (1990). The result is noted for the non-local relationship between the two quantities; the inverse Fourier transform is an integral over all space. This is considered an essential difference between the collisional and collisionless theory, and is due to the free-streaming effect in the collisionless regime.

#### Regulation of the Heat Flux by Wave-Particle Interactions

The framework of quasilinear theory describes the effect of wave–particle interactions in the modification of the heat flux $\boldsymbol{q}_{s}$ of species s as a diffusion of the velocity distribution function in velocity space (Kennel and Engelmann 1966; Verscharen et al. 2019a). In a magnetised plasma, resonant wave–particle interactions are effective if the participating waves and particles fulfill the resonance condition

30 $$ \omega _{\mathrm {r}}-k_{\parallel}v_{\parallel}=p\Omega _{s}, $$

where $\omega _{\mathrm {r}}$ is the real part of the wave frequency, $k_{\parallel}$ is the component of the wave vector parallel to the background magnetic field $\boldsymbol{B}_{0}$, $v_{\parallel}$ is the component of the particle velocity parallel to $\boldsymbol{B}_{0}$, p is the integer order of the resonance, and $\Omega _{s}=q_{s}B_{0}/m_{s}c$ is the gyro-frequency of species s. The resonance with p=0 is called *Landau resonance*, while all resonances with p≠0 are called *cyclotron resonances*.

The resonance condition in Eq. (30) is a necessary but not a sufficient condition for quasilinear diffusion. In addition to the fulfilment of Eq. (30), constraints on the polarisation of the involved wave exist. For instance, waves propagating parallel to $\boldsymbol{B}_{0}$ can only participate in cyclotron resonances if they have a right-handed (p=−1) or left-handed polarisation (p=+1). Likewise, waves propagating parallel to $\boldsymbol{B}_{0}$ can only participate in Landau resonance (p=0) if the wave electric field has a finite component parallel to $\boldsymbol{B}_{0}$, for the mathematical derivation of these conditions, see Verscharen et al. (2019a).

As the velocity distribution function diffuses in velocity space, the total kinetic energy of the particles changes. If the particles gain kinetic energy in this process, energy conservation demands that this energy is taken from the resonant waves. The amplitude of the resonance waves thus decreases, which corresponds to wave damping. If instead the particles lose kinetic energy in this process, energy conservation demands that this energy is given to the resonant waves. The amplitude of the resonant waves thus increases, which corresponds to wave instability. In both cases, the quasilinear diffusion modifies the total kinetic energy of the particles, and, in general, also the heat flux as the third moment of the distribution. In those cases, wave–particle interactions change the heat flux and thus contribute to the energy balance.

**Fig. 8:**
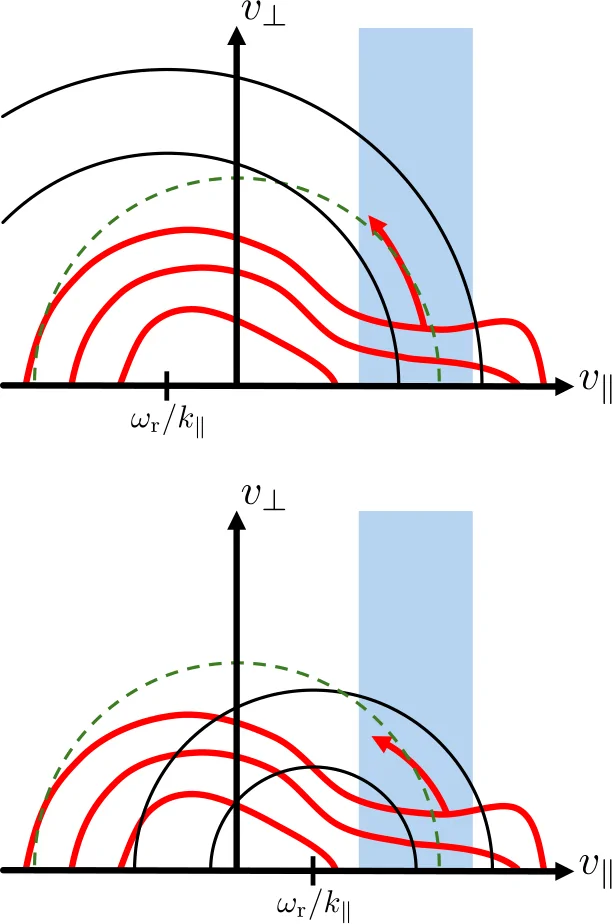
Illustration of the regulation of heat flux by resonant wave–particle interactions. The red contours represent iso-contours of the velocity distribution function. The black circles are centred around $\omega _{\mathrm {r}}/k_{\parallel}$. The red arrow shows the local direction of diffusive flux of resonant particles in velocity space. The blue shaded area indicates the assumed range of velocities that fulfils Eq. (30). The green dashed circle indicates a contour of constant kinetic energy. The top panel represents a situation of wave damping; the bottom panel shows a situation of wave growth

Figure 8 illustrates the change of heat flux through resonant wave–particle interactions. The individual panels show two-dimensional velocity space, where $v_{\perp}$ is the component of the velocity perpendicular to $\boldsymbol{B}_{0}$ and $v_{\parallel}$ is the component of the velocity parallel to $\boldsymbol{B}_{0}$. The red contours represent iso-contours of the assumed velocity distribution in this case. This distribution has a finite heat flux in the form of its skewness towards larger $v_{\parallel}$. Moreover, we assume the presence of a plasma wave with real frequency $\omega _{\mathrm {r}}$ and wavevector $\boldsymbol{k}$, and we assume that only particles in the blue shaded area fulfill the resonance condition in Eq. (30) for cyclotron resonance as well as the required associated polarisation relations.

Under these conditions, quasilinear theory predicts that the resonant particles diffuse in velocity space. The diffusive flux of particles is locally tangent to semi-circles centred around $\omega _{\mathrm {r}}/k_{\parallel}$ (shown in black). At the same time, the diffusive flux is directed from larger values of the distribution function towards smaller values of the distribution function. Therefore, we predict diffusion as indicated by the red arrows in Fig. 8.

Depending on the shape of the velocity distribution function, the sign of $\omega _{\mathrm {r}}/k_{\parallel}$, and the location of the resonant particles in velocity space, this type of interaction leads to either a gain in kinetic energy or a loss of kinetic energy of the resonant particles. The green dashed circle in Fig. 8 indicates a contour of constant kinetic energy (i.e., $v_{\perp}^{2}+v_{\parallel}^{2}=\mathrm{constant}$). The configuration in the top panel of Fig. 8 leads to a gain in kinetic energy of the resonant particles, as indicated by the relative alignment between the red arrow and the green dashed circle. Correspondingly, the configuration in the bottom panel leads to a loss of kinetic energy of the resonant particles. Therefore, the configuration in the top panel illustrates a case of wave damping, and the configuration in the bottom panel illustrates a case of wave instability. In both cases, however, the wave–particle interactions modify the heat flux of the distribution by increasing $v_{\perp}$ and reducing $v_{\parallel}$ of the resonant particles. This is the essence of the collisionless heat-flux modification through resonant wave–particle interactions.

In the case of wave damping or instability, the wave frequency is complex with a non-zero imaginary part, $\omega =\omega _{\mathrm {r}}+i\gamma $ (Kennel and Wong 1967). The wave amplitude A then changes exponentially in time according to $A\propto e^{\gamma t}$. Consequently, wave damping requires the presence of waves with sufficient amplitude in the plasma that then undergo wave–particle interactions in the described way. In the case of wave instability, the amplitude of the unstable waves grows exponentially out of the thermal noise of electric and magnetic fluctuations, fed by the energy of the resonant particles. Therefore, instabilities are of particular interest in the context of heat-flux regulation, as they only require the presence of an appropriate velocity distribution function to trigger their growth.

Many families of resonant instabilities exist that are driven by electron heat flux (Verscharen et al. 2022a). Fully kinetic simulations of a subset of these instabilities are presented, e.g., by Komarov et al. (2018), Roberg-Clark et al. (2018a), Micera et al. (2020), and Micera et al. (2021). These simulations confirm that the underlying physics of resonant instabilities driven by electron heat flux is in general described by the quasilinear framework introduced above.

For statistical studies, it is often beneficial to work with closed representations of the instability thresholds that depend on the key plasma parameters. Equation (10) is such a representation; we rewrite the equation here for convenience

31 $$\begin{aligned} \hat{q}^{\mathrm{e}}_{\mathrm{inst}}(S_{q}, a_{q}, \alpha _{q}) = \frac{1}{q_{\mathrm{sat}}} \frac{S_{q}}{(\beta _{\mathrm{e}} + a_{q})^{\alpha _{q}}}. \end{aligned}$$

The hat ($\hat{q}^{\mathrm{e}}_{\mathrm{inst}}$) indicates normalisation to the free-streaming value. Useful parameters are reported in Table 2. The values used for Vasko et al. (2019) are calculated for instability, therefore these contours indicate a maximum growth rate, normalised to the absolute value of the electron gyrofrequency ($\gamma / |\Omega _{\mathrm{e}}|$), in the $\hat{q}_{\parallel}^{\mathrm{e}}$-$\beta _{\mathrm{e}}$ space. Typically, thresholds are calculated for a value of $\gamma / |\Omega _{\mathrm{e}}|$ (Table 3), so that when $\hat{q}_{\parallel}^{\mathrm{e}} \geq \hat{q}^{\mathrm{e}}_{ \mathrm{inst}}$, the system is unstable. The value of $\gamma / |\Omega _{\mathrm{e}}|$ is chosen based on the physical system, for example Vasko et al. (2019) chooses values relevant to average solar wind observations. The semi-empirical line derived by Gary et al. (1994) is an empirical fit to observations from the Ulysses spacecraft, therefore their relationship is not at constant $\gamma / |\Omega _{\mathrm{e}}|$ in the $\hat{q}_{\parallel}^{\mathrm{e}}$-$\beta _{\mathrm{e}}$ space but requires another parameter (e.g. properties of the beam temperature anisotropy), where it might be a contour for constant $\gamma / |\Omega _{\mathrm{e}}|$. Pistinner and Eichler (1998) take on a different approach, where they directly solve a simplified kinetic equation for the velocity distribution function, and then calculate γ and $\hat{q}_{\parallel}^{\mathrm{e}}$ directly. Their analysis leads to a relation of the form Eq. (31).

**Table 3:** Parameters used in the calculation of instability thresholds that appear in Fig. 9 according to Eq. (22) (repeated here as Eq. (31))

Parameters in Fig. 9					
Reference	$S_{q}$	$a_{q}$	$\alpha _{q}$	$\Delta _{s}/v_{\mathrm{th}}^{\mathrm{c}}$	$\gamma /|\Omega _{\mathrm{e}}|$
Vasko et al. (2019)	0.2	0.25	0.5	0	5 × 10^−4^
Vasko et al. (2019)	0.22	0.24	0.52	0.5	5 × 10^−4^
Vasko et al. (2019)	0.26	0.19	0.53	1.0	5 × 10^−4^
Vasko et al. (2019)	0.33	0.13	0.6	1.5	5 × 10^−4^
Vasko et al. (2019)	0.39	0.11	0.63	2.0	5 × 10^−4^
Gary et al. (1999)	1.0	0.0	0.82	N/A	N/A
Pistinner and Eichler (1998)	2.6/3$\sqrt{\pi}$	0.0	1.0	N/A	N/A

**Fig. 9:**
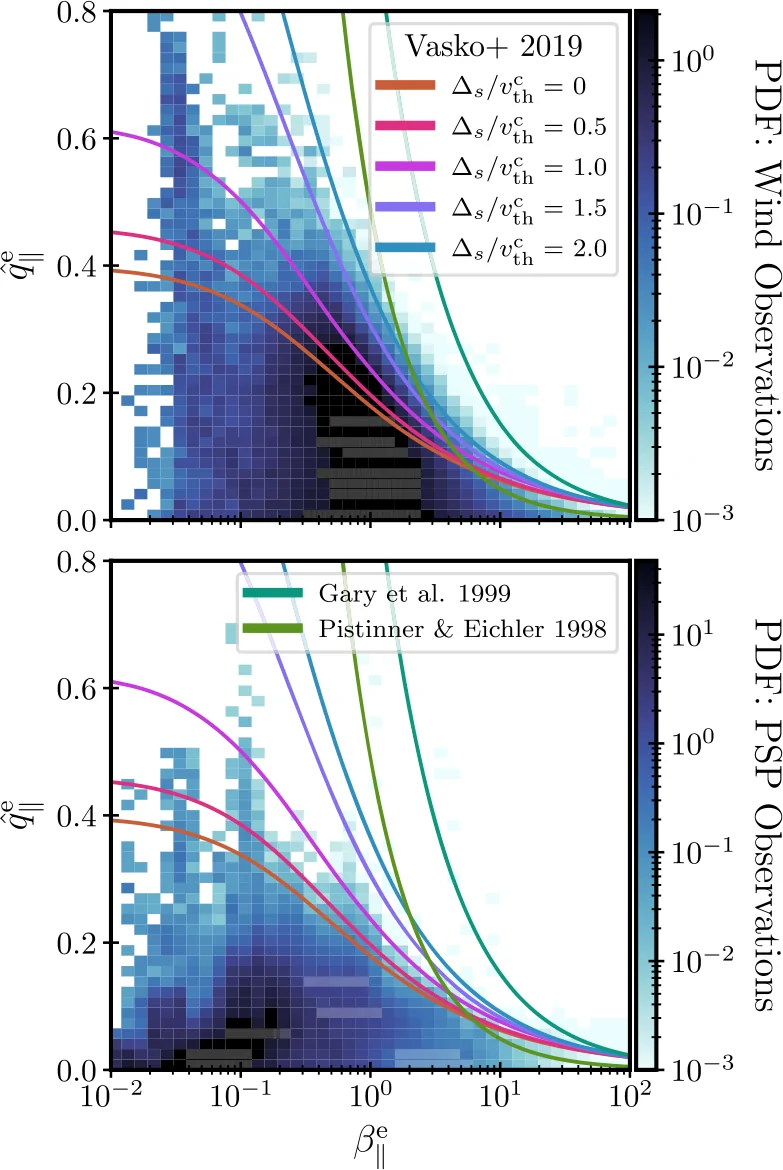
Normalized parallel heat flux measurements from Wind (top) and Parker Solar Probe (bottom). The observations are described in Sect. 1.4. The coloured curves represent parametric instability thresholds from Eq. (10) with parameters given in Table 3. The legends are global and are split over the top two panels of the figure

In Fig. 9, we display the normalised heat flux from measurements from two spacecraft: Wind and Parker Solar Probe. We note that the vertical axis differs by an order of magnitude between for first and second panel. We emphasise that parametric representations such as Eq. (31) with parameters in Table 3 are strictly only valid for a given assumed shape of the background distribution and over a defined range of plasma parameters.

It is often easier in numerical plasma models to compose the total electron distribution as the sum of individual (bi-)Maxwellian electron populations than creating a complex one-population distribution. Therefore, numerical plasma models of heat-flux instabilities are often created by considering at least two electron populations: a core population that dominates the density and a beam that determines the heat flux of the overall electron distributions.

#### The Moments of the Quasilinear Equations

Linear theory provides information on the stability on the VDF; when the growth rate is large, these configurations are interpreted as thresholds. However, linear theory does not provide information on the saturated state of the system. Quasilinear theory does provide further information on the saturated states, but formidable complexity arises when attempting to derive a transport theory. In order to investigate this further, we briefly introduce the so-called moment quasilinear theory in order to demonstrate the difficulty of a kinetic transport theory.

Quasilinear theory decomposes the VDF into a homogeneous background, that evolves slower than the cyclotron period, and a perturbation (Marsch and Tu 2001; Marsch 2006). The solution to the perturbation is assumed to be linear, and is precisely the linear theory result. The background VDF evolves according to the quadratic of the linear perturbation and the energy spectrum of the magnetic field. This framework describes the changes of the background VDF due to resonant-diffusion. Two notable assumption are: 1) the magnetic field does not interact non-linearly, 2) the growth rates are weak e.g., $\gamma / |\Omega _{\mathrm{s}}| \ll 1$. The quasilinear evolution is a reliable approximation for the description of resonant wave–particle interactions until particle trapping becomes relevant (Verscharen et al. 2026).

Quasilinear theory provides self-consistent differential equations that describe how the background VDF, which we call $F_{\mathrm{s}}$ and the magnetic field spectrum evolve. It is then straightforward to take moment of the evolution equation for $F_{\mathrm{s}}$, see reviews by Yoon (2017) and Marsch (2006).

The first, second, and third moment of the time-evolution equation for the background VDF produces

32 $$\begin{array}{c} \frac{1}{n_{s}}\frac{\partial }{\partial t}\left(\begin{array}{c} n_{s}u_{∥}^{s}\\ p_{∥}^{s}\\ p_{⊥}^{s}\\ q_{∥}^{s}\\ q_{⊥}^{s} \end{array}\right)=\frac{\Omega_{s}^{2}}{{\left(2\pi \right)}^{3}}\int_{-\infty }^{\infty }d^{3}k\;\hat{B}(k)\frac{1}{k_{∥}}\sum_{p_{r}=-\infty }^{\infty }\int_{0}^{\infty }dv_{⊥}\;R_{s}(k,p_{r},v_{⊥})\\ \times \left(\begin{array}{c} 1\\ 2m_{s}V_{s}^{\prime }(k,p_{r})\\ \frac{p_{r}m_{s}\Omega_{s}}{k_{∥}}\\ 3m_{s}V_{s}^{\prime }{\left(k,p_{r}\right)}^{2}\\ \frac{m_{s}}{2}(v_{⊥}^{2}+2V_{s}^{\prime }(k,p_{r})\frac{p_{r}\Omega_{s}}{k_{∥}}) \end{array}\right)+\left(\begin{array}{c} 0\\ 0\\ 0\\ -3\frac{p_{∥}^{s}}{n_{s}}\frac{\partial u_{∥}^{s}}{\partial t}\\ -\frac{p_{⊥}^{s}}{n_{s}}\frac{\partial u_{∥}^{s}}{\partial t} \end{array}\right), \end{array}$$

where the zeroth moment ($n_{s}$) is constant. For brevity, we only introduce the quantities qualitatively. The resonance function ($\mathcal{R}_{s}({ \mathbf{k}}, p_{r}, v_{\perp})$) contains information on waveframe pitch-angle cyclotron (order denoted by $p_{r}$) resonant interaction. The quantity $\hat{\mathcal{B}} \big ( {\mathbf{k}} \big)$ is proportional to the normalised magnetic field energy spectrum and accounts for wave ellipticity and field parallel power. For the case of parallel propagating circularly polarised waves, $\hat{\mathcal{B}} \big ( {\mathbf{k}} \big)$ is the normalised energy spectrum. The Doppler shifted resonant speed,

33 $$\begin{aligned} V'_{s} ({\mathbf{k}}, p_{r}) = \frac{\omega ({\mathbf{k}}) - p_{r}\, \Omega _{s}}{k_{\parallel}} - u_{ \parallel}^{s} k_{\parallel}, \end{aligned}$$

where $\omega ({\mathbf{k}})$ is the real part of the dispersion relation. The evolution of the moments requires knowledge of the magnetic energy spectrum and the wave-particle resonance, both of which evolve in time, creating severe difficulty in producing a useful set of equations (Dum 1978b,a).

As a simplification to this complex framework, some models demand that the distribution function follows a prescribed functional shape, e.g., a drifting bi-Maxwellian, while the underlying function parameters evolve according to quasilinear theory (Yoon 2017). This is a closure, as high-order moments, such as the heat flux, are described by the parameters of the assumed VDF, which are low-order moments.

The basic premise of this approach is to solve the time-dependent moments of the quasilinear equations, as per Eq. (32), while forcing $F_{s}$ to obey the model distribution function. With a simplified expression for the dielectric tensor and thus the dispersion relation, the evolution of the moments and the magnetic energy follow differential equations which can be solved with basic numerical methods.

Early work with this method targets ion instabilities driven by temperature anisotropy (Davidson and Ogden 1975), the isotropisation of bi-Maxwellian and loss-cone distributions through whistler-wave instabilities (Ossakow et al. 1972), or electron and ion pinch experiments (Davidson 1972). With relevance to electron transport, this work suggests that the relaxation time of the distribution function is much shorter than time scale of which Coulomb collisions isotropise the distribution (Davidson 1972). This quasilinear moment approach is also used for studies of electron physics in the solar wind (Ali et al. 2020; Sarfraz et al. 2022; Yoon et al. 2024).

The incorporation of quasilinear relaxation into transport theory has been attempted with some success, see e.g. Husidic et al. (2022) where transport coefficients are calculated for non-equilibrium, suprathermal plasmas. The marginal stability approach reduces this problem to identifying a growth rate from some unstable configuration and adding the so-called anomalous growth to the evolution equations for the temperatures and densities (Manheimer and Boris 1977). The problem is severely complicated by two issues: (1) transport coefficients cannot be specified by local fluid parameters alone, and (2) the electromagnetic spectrum is dynamic on the relevant timescales (Dum 1978b,a). It is possible to incorporate the relaxation due to quasilinear diffusion, often called *effective collisions* (Dum 1978a). This approach provides effective collision frequencies due to wave–particle interactions that are greater than Coulomb collision frequencies (Yerger et al. 2025). We discuss effective collision operators in more detail in Sect. 4.

### Bridging the Collisionless and Collisional Closures

In this section we review two classes of closures that attempt to bridge the collisionless and collisional regimes. Most of them are formulated to account for the electron heat flux. The first class explore the velocity space dependence of collisional operators. The second class attempt to bridge the results with solely fluid moments.

#### Velocity Space Closures

Here, we review an important class of closures that accounts for the velocity dependence of the collision operator. One can consider the so-called deflection time-scale for self-collisions from Spitzer and Härm (1953), that establishes isotropy for a low-order perturbation of the distribution function

34 $$\begin{aligned} \hat{\nu}_{s,s}(\hat{v}_{s}) = \frac{(2 \hat{v}_{s}^{2} - 1) \phi (\hat{v}_{s}) + \hat{v}_{s} \phi '(\hat{v}_{s})}{2 \hat{v}_{s}^{5}} \frac{\Gamma _{s}}{v_{\mathrm{th,s}}^{3}} \end{aligned}$$

with $\hat{\nu}_{s,s}$ the velocity dependent collision frequency and $\hat{v}_{s} = |{\mathbf{v}}|/v_{\mathrm{th,s}}$. The $\phi (\hat{v})$ is the error function with derivative $\phi '(\hat{v})$, and $\Gamma _{s} = 8 \pi q_{s}^{4} n_{s} \mathrm{ln}|\Lambda _{s}|/m_{s}^{2}$ where $\mathrm{ln}|\Lambda _{s}|$ is as before the Coulomb logarithm. Notice that for $\hat{v}_{s} \gg 1$ the function $\hat{\nu}_{s,s}(\hat{v}_{s}) \propto \hat{v}_{s}^{-3}$, so that time it takes to restore isotropy increases for large $\hat{v}_{s}$ (Scudder and Olbert 1979). Marsch and Goldstein (1983) study this effect in the solar wind, and show that in velocity space both a collision dominated and collisionless regime exist.

In Scudder (1992), it is assumed that the distribution function in a collision-dominated plasmas is a Lorenztian or kappa distribution in the small $\hat{v}_{s}$ regime, and a power law in $\hat{v}_{s}$ in the suprathermal regime. Scudder (1992) then solves for the distribution function subject to a conservative decelerating potential and shows that the temperature of the distribution can increase while the density decreases. These conditions are analogous to the solar coronal transition region, and so support a corona heating mechanism.

Pistinner and Eichler (1998) study the scenario where collisions dominate small $\hat{v}_{s}$ and whistler turbulence dominates large $\hat{v}_{s}$. They study a simplified Vlasov-Boltzmann equation and make the ansatz that the anisotropic Lorentzian distribution function is the solution. The linear stability of the solution is studied to find conditions when temperature gradients will make the plasma unstable to whistler waves. The resulting quasi-linear diffusion causes self-inhibition of the electron heat flux (Roberg-Clark et al. 2018a; Komarov et al. 2018; Drake et al. 2021; Yerger et al. 2025). This is likely a more appropriate understanding of moderate electron beta $\beta _{\mathrm{e}} > 10^{-1}$ and non-small Knudsen number, as derived by Pistinner and Eichler (1998) Kn $\geq 2 \times 10^{-4}$, opposed to the assumptions made in the Braginskii fluid equations.

Bott et al. (2024) has made considerable developments on the stability of weakly collisional high-beta plasmas and provides a thorough review of literature on the topic. They employ the usual Chapman-Enskog (CE) approach, by noting that at linear order, the collisional terms contribute coefficients that depend on $|{\mathbf{v}}| /v_{\mathrm{th,e}}$, so that the total distribution is of the form

35 $$\begin{aligned} f^{\mathrm{CE}}_{\mathrm{e}} = f_{\mathrm{e}}^{\mathrm{M}} \; \bigg\{ 1 + \text{1st order CE terms} \bigg\} , \end{aligned}$$

This is the so-called Chapman-Enskog equilibrium, which is a closure of the fluid equations. The $f_{\mathrm{e}}^{\mathrm{M}}$ is the Maxwellian distribution function, and the “1st order CE terms” contain contributions from temperature and bulk velocity perturbations. Bott et al. (2024) then studies the stability of the equilibrium subjected to the linear Vlasov-Maxwell equations to produce the thresholds that appear in Fig. 1. The generality of these findings by Bott et al. (2024) beyond solely the whistler wave confirms that the classical Braginskii regime is still recovered in the strongly magnetized and low beta regime, but in the high beta and strongly magnetized regime, collisional regulation is always subdominant to collisionless microinstabilities.

#### Moment-Based Closures

Collisional closures are based on a low-order expansion of $\lambda _{\mathrm{mfp}}^{\mathrm{s,s}}/L_{T_{\mathrm{s}}}, \; \rho ^{ \mathrm{s}}/L_{T_{\mathrm{s}}}$. Hence, one would expect that collisional closure approach the collisionless regime when collisionality decreases.

This is however not the case because the definition of $\lambda _{\mathrm{mfp}}^{s}$ is based on a low-order expansion of the distribution function itself. This notwithstanding, the Landau fluid equations recover some similarities to Braginskii’s fluid equations, for example, the relation between the parallel heat flux and the parallel electron temperature. The incompatibility can be appreciated by the noticing Eq. (21) diverges for small $\nu _{\mathrm{e,e}}$.

A thorough comparison of linear solutions to high-order Braginskii-fluid equations and Landau fluid equations appears in Fig. 2 of Hunana et al. (2022) where even a 16-moment closure based on expansion about a bi-Maxwellian fails to recover any effects similar to Landau damping near $k_{\parallel} \lambda _{\mathrm{mfp}}^{\mathrm{e}} \gtrsim 1$. There have been numerous attempts at bridging closures of the electron heat flux between the collisional and collisionless regimes.

The general form of these closures is

36 $$\begin{aligned} q_{\mathrm{bridge}} = \frac{\nu _{\mathrm{ratio}} \, q_{\mathrm{collisionless}} + q_{\mathrm{collisional}}}{\nu _{\mathrm{ratio}} + 1} \end{aligned}$$

where $q_{\mathrm{bridge}}$ bridges the collisionless heat flux $q_{\mathrm{collisionless}}$ and the collisional heat flux $q_{\mathrm{collisional}}$ with the parameter $\nu _{\mathrm{ratio}}$, where the parameter depends on fluid moments. This property makes the adaption of these closures much easier, with respect to velocity-space dependent versions. The parameter $\nu _{\mathrm{ratio}}$ is typically proportional to relaxation frequencies of the two regimes, and can be modified to that it approaches values of 0 and ≫1 in the desired limits. This approach was used by Cranmer et al. (2007), used for demonstrative purposes in this article, in particular in Figs. 1, 2, and 7. Cranmer and Schiff (2021) recently explored more of these types of closures with relevance to the solar wind.

Typically the collisional heat flux is taken to be Spitzer-Härm heat flux,

37 $$\begin{aligned} q^{\mathrm{SH}}_{\parallel \mathrm{e}} = - \kappa _{\parallel , T}^{ \mathrm{e}} \frac{p_{\mathrm{e}}}{m_{\mathrm{e}} \nu _{\mathrm{e,e}}} \frac{\partial}{\partial x_{\parallel}} T_{\mathrm{e}} \end{aligned}$$

where the ∥ subscript is parallel to the local magnetic field. Cowie and McKee (1977) attempted to bridge the collisional Spitzer-Härm value to the saturation heat flux with the following formula

38 $$\begin{aligned} q_{\parallel , \mathrm{e}}^{\mathrm{C \& M, 1977}} \approx \frac{1}{2} m_{\mathrm{e}} n_{\mathrm{e}} v_{\mathrm{th, e}}^{3} \frac{\lambda _{\mathrm{mfp}}^{\mathrm{e}}}{L_{\mathrm{e}} + 4 \lambda _{\mathrm{mfp}}^{\mathrm{e}}}. \end{aligned}$$

Cowie and McKee (1977) show that the saturation heat flux value results from assuming a Maxwellian source subjected to an infinite temperature gradient with zero current and where stability is maintained.

Komarov et al. (2018) present the following formula

39 $$\begin{aligned} q_{\parallel , \mathrm{e}}^{\mathrm{K, 2018}} \approx \frac{1}{2} m_{ \mathrm{e}} n_{\mathrm{e}} v_{\mathrm{th, e}}^{3} \; \frac{1}{1/\mathrm{Kn} + \beta _{\mathrm{e}}/3 + 4 } \end{aligned}$$

which bridges the collisional heat flux and the heat flux suppressed by microinstabilities, $q_{\parallel , \mathrm{e}} \sim 1 / \beta _{\mathrm{e}}$ in the collisionless regime (cf. Roberg-Clark et al. 2018a). In attempt to achieve the same asymptotic heat fluxes, Perrone et al. (2024b) write

40 $$\begin{aligned} q_{\parallel , \mathrm{e}}^{\mathrm{P,2024}} \approx 3 \frac{q_{\mathrm{e}}^{\mathrm{SH}}}{3 + \beta _{\mathrm{e}} \,\mathrm{Kn} }. \end{aligned}$$

which also smoothly interpolates between the collisional and collisionless regimes.

In Fig, 10 we compare these theoretical values to solar wind measurements. While short regions of parameter space indicate some validity, in general, very few of the observations can be accounted for.

**Fig. 10:**
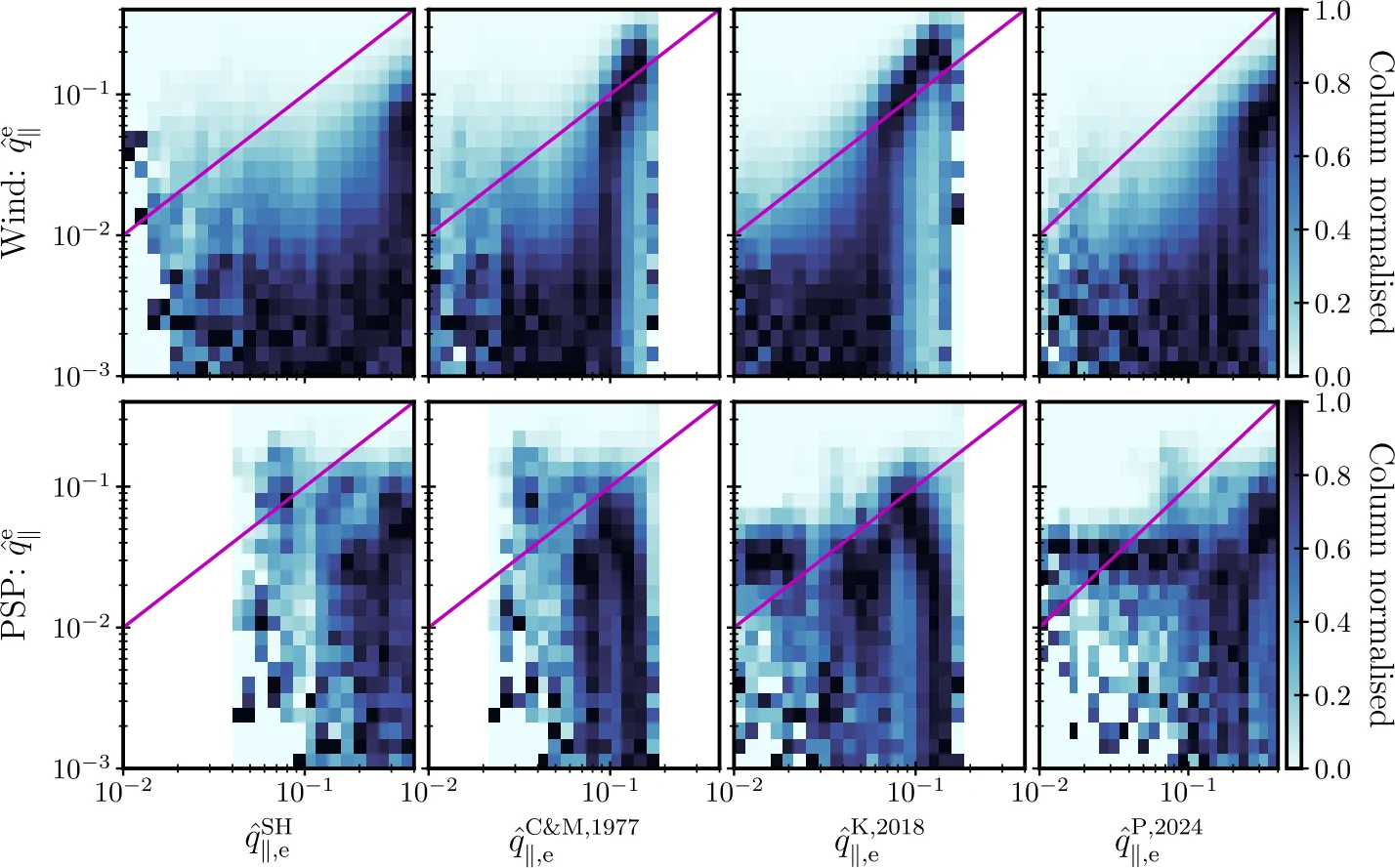
Comparison of heat flux closures (horizontal axis) to measurements (vertical axis) from Wind (top panels) and PSP (bottom panels). These datasets are explained in Sect. 1.4. The theoretical heat fluxes that appear on the horizontal axes are well-defined in the text. The magenta line is equivalence between the two expressions. The data is binned so that the columns are normalised by the column maximum value

These heat flux prescriptions are particularly useful as heat flux closures in simulations of large-scale systems, where kinetic simulations are unfeasible. Beckmann et al. (2022) study a variety of these heat flux prescriptions in the context of the cooling flow problem in active galactic nulcei induced by the dominance of the heat buoyancy instability. They find that the suppressed heat flux in the collisionless regime is unable to prevent the heat buoyancy instability. Perrone et al. (2024b) investigate the effect of collisionless suppression of thermal conductivity on the magneto-thermal instability, finding that beyond a critical level of suppression the system is not able to sustain turbulent fields, which would be incompatible with observations. These works demonstrate the need for a better understanding of microscale phenomena and how it feeds back on the macro scales of the system (Kunz et al. 2011).

## Derivation of Heat-Flux Closures from Collisionless Simulations

The modification of the electron velocity distribution function and related heat flux evolution due to collisionless processes can be simulated from first principles using fully kinetic simulations, as done e.g. in Roberg-Clark et al. (2018a), Kuzichev et al. (2019), Micera et al. (2020, 2021, 2025), Kuzichev et al. (2023), Yerger et al. (2025) for heat flux regulation by whistler instabilities. The drawback of a fully kinetic description is of course computational cost, which limits applicability to large-scale problems. Significant effort has been dedicated in recent years to the task of finding a relatively simple form for the heat flux closure that could be used to close multifluid 10-moment models. Since 10-moment models evolve particle moments up to 6 unique components of the pressure tensor included, they are considered a good compromise between low computational cost and physical expressivity. One common strategy is to use fully kinetic simulations as the “ground truth”, and identify relatively simple forms for the heat flux closure parametrized on simulation parameters and requiring only information available in 10-moment simulations, such as electric and magnetic fields and lower moments. The form of the closure to parametrize is guided by physical considerations.

Equations (10) (theoretical maximum threshold for instabilities) and (11) (Hollweg formulation of heat flux for free streaming plasmas) described above provide great examples of this strategy: physics informs the choice of the form of the closure, and free parameters can be derived from either observations or simulations. However, Eq. (10) provides maximum threshold values, rather than a prediction accurate enough for the electron species in 10-moment simulations. Similarly, Eq. (11) is preferentially used in extremely large scale simulations, such as heliospheric-wide simulations where the focus is more on proton- or system-scale dynamics (a very incomplete list includes e.g. Chandran et al. 2011; Holst et al. 2014; Réville et al. 2020), rather than in more targeted simulations requiring an accurate description of electron-scale processes. Incidentally, in the large-scale model described above Eq. (11) models heat flux regulation by collisionless processes, and a transition to a collisionally regulated heat flux is prescribed at lower heliocentric distances (see Sect. 3.3).

In this section, we will summarize recent efforts aimed at obtaining a heat flux closure for collisionless processes from fully kinetic simulations. We divide these works in four categories

- derivation of a scalar effective scattering frequency
- derivation of effective collisional operators
- parametrization of Landau-fluid closures
- application of Machine Learning to the closure problem

### Calculation of Effective Scattering Frequency from Collisionless Processes

The assumption underlying this first class of work is that the Braginskii formulation for the parallel heat flux in collisional plasmas

41 $$ q_{\parallel , \mathrm{Brag}}= - \dfrac{3}{2} n \dfrac{v^{2}_{th,\parallel}}{\nu _{eff}} \nabla _{\parallel }T_{ \parallel } $$

can be extended to heat flux evolution by collisionless processes by recasting the collisional frequency $\nu _{eff}$ as the effective scattering rate^3^ associated with particle scattering by the collisionless process under investigation (Kunz et al. 2020).

Strategies for the calculation of effective scattering rates from collisionless processes are identified and applied in Kunz et al. (2014) and in the follow-up studies Melville et al. (2016), Kunz et al. (2020), Bott et al. (2021) to *ion* scattering processes, namely firehose and mirror instabilities. Since the same strategies are applicable to *electron* scattering processes, they will be described here.

In Kunz et al. (2014) shearing box hybrid simulations are used to drive the plasma unstable to either ion firehose or mirror instabilities by respectively decreasing and increasing the magnetic field magnitude. A complementary approach, the Expanding Box approach (Grappin and Velli 1996) has been similarly used to trigger ion and electron firehose instabilities, and can be used for the triggering of ion cyclotron, mirror and whistler instabilities respectively for protons and electrons by turning expansion into compression (Ahmadi et al. 2017). Due to the shearing, a pressure anisotropy develops, with $p_{\perp }/ p_{\parallel}$ decreasing/ increasing with decreasing/ increasing magnetic field magnitude as a consequence of conservation of the first adiabatic invariant $\mu := m_{i} v_{\perp}^{2}/ 2B$. The measurement of the instability-driven scattering rate relies on the expectation that the first adiabatic invariant is conserved for most particles during the CGL-like phase of simulation evolution, before instabilities set in. After instabilities are triggered, due to crossing of the respective instability thresholds, proton scattering by firehose and mirror fluctuations respectively are expected to pin pressure anisotropy during saturation phase to instability threshold. The scattering rate is measured by tracking a (relatively low) number of randomly selected particles, constructing a distribution of the times it takes to each of them to change its μ of a factor e, and taking the width of the resulting exponential factor to be $1/ \nu _{eff}$. In firehose instability simulations, a comparison is then made between the scattering frequency $\nu _{eff}$ so obtained and the scattering frequency $\nu _{f}$ needed to maintain pressure anisotropy at firehose instability threshold, $\Lambda _{f}:=1- p_{\perp}/ p_{\parallel }-2 / \beta _{\parallel }=0$. Quite interestingly, the relation used to relate scattering rate ν and pressure anisotropy, $p_{\perp}/ p_{\parallel }-1 = (3/ \nu ) (d\; \mathrm{ln} | \langle \mathbf{B} \rangle |/ dt)$ was obtained for *collisional*, incompressible plasmas without heat flow (Rosin et al. 2011). One can verify that $\nu _{eff} \sim \nu _{f} := 3 (\beta _{\parallel , sat}/ 2) (d \; \mathrm{ln}\; | \langle \mathbf{B} \rangle |/dt)_{sat} $. In mirror instability simulations it is similarly verified that $\nu _{eff} \sim \nu _{m}$, where $\nu _{m}= 3 \beta _{\perp , sat} (d \mathrm{ln} | \langle \mathbf{B} \rangle |/ dt)_{sat}$ is the scattering required to maintain pressure anisotropy at instability threshold values. It is observed that scattering rates are significantly different for trapped and passing particles.

Similar strategies for the calculations of the effective scattering rates are applied to *electrons* in simulations of whistler heat flux instability in high-beta plasmas in Roberg-Clark et al. (2018a), Komarov et al. (2018). The aim is to explore electron scattering by whistler heat flux instability as a way of suppressing parallel heat flux below Spitzer-Harm levels in the weakly magnetized, collisionless plasmas in the intracluster medium. In both sets of fully kinetic PIC simulations, the measured parallel heat flux is found to scale with $1/ \beta _{e}$ (as expected already from the quasilinear derivation in Pistinner and Eichler 1998) rather than with $\nabla T_{e}$, as in collisional plasmas. The scattering rate $\nu _{\mathrm{eff}}$ (or, equivalently, the scattering time) is calculated by tracing electron trajectories in the simulations.

These results are relevant under several points of view. First, they show that at least some collisionless processes can be understood and modeled by minimally extending frameworks developed for collisional plasmas. Turbulence in collisionless and semi-collisional plasmas seems to be another process where collisional-like quantities, such as effective viscosity, resistivity and Reynolds numbers can be obtained from both PIC simulations and spacecraft observations (Bandyopadhyay et al. 2023b; Yang et al. 2024). An interesting open question is whether only certain classes of collisionless processes are amenable to this simplified fluid-like description and, if so, which characteristics they share. Second, they show relations can be found which tie plasma parameters (here, magnetic field value and beta at saturation) to effective scattering rate and hence, potentially, to heat flux, thus opening interesting perspectives for modeling. However, a very relevant point is raised by Bott et al. (2021) when studying the role of proton firehose instabilities in an expanding hybrid simulation of strongly driven Alfvénic turbulence: a velocity distribution function modified by collisionless processes (for example, the VDF at saturation of a kinetic instability) differs significantly from one processed by ‘real’ collisions. In particular, in the case of proton firehose instability, Bott et al. (2021) compares the characteristic ‘butterfly’ shape with the mildly anisotropic VDF one would expect after saturation by collisional processes. This consideration points to the appropriateness of deriving from simulations effective collisional operators which depends on the position of the system in phase space.

#### Anomalous Resistivity at Shocks

We now describe an exercise in the estimation of anomalous resistivity and effective collisionality by ion acoustic (hence, *ion*-driven) waves in collisionless shocks. This ion-focused section is present in this review on electron transport for two reasons: first, effective collisions from ion waves can affect electron transport at shocks; second, this is a rare case where effective transport coefficients calculated in observations and simulations are compared.

When estimating transport coefficients, Sagdeev (1966), Vedenov (1963) introduced a quasi-linear approximation for the effective drag force produced by fluctuating electromagnetic fields, which was called an *anomalous resistivity*, $\eta{_{wave}}$, because it acted on the current-carrying particles. An effective collision frequency between wave fields and particles, $\nu{_{wave}}$, was estimated, which describes the rate of energy/momentum transfer between electromagnetic fields and charged particles. This transfer can be irreversible and is therefore analogous to an effective friction or drag force. The analytical form of $\nu{_{wave}}$ depends on the dispersion relation for the wave mode that produces these so-called wave-particle interactions. One of the first analytic forms for $\nu{_{wave}}$ and $\eta{_{wave}}$ was calculated for ion acoustic waves (IAWs), due to their relatively simple dispersion relation:

42a $$\begin{aligned} \nu{_{iaw}} & = \omega{_{pe}} \frac{\varepsilon{_{o}} \mid \delta E \mid ^{2}}{2 n{_{e}} k{_{B}} T{_{e}}} \end{aligned}$$

42b $$\begin{aligned} \eta{_{iaw}} & = \frac{ \nu{_{iaw}} }{\varepsilon{_{o}} \omega{_{pe}}^{2}} \end{aligned}$$

where δE is the fluctuating electric field amplitude of the wave. Note that formally η should be a resistivity tensor. Thus, fluctuating electric fields can affect both currents and heat flux.

In a statistical study of electrostatic waves near collisionless shocks (Wilson et al. 2007) it was found that IAWs could generate anomalous resistivities of $\eta{_{iaw}}$ ∼ 1–860 $\Omega \ m$. If we compare this range to the values estimated from Coulomb collisions, we find $\eta{_{iaw}}/\eta{_{ee}}$ ∼ $10^{3}$–$10^{8}$. A more recent study of waves at the terrestrial bow shock (Wilson et al. 2014a,b) found $\eta{_{iaw}}$ above $10^{4}$ $\Omega \ m$, corresponding to $\eta{_{iaw}}/\eta{_{ee}}$ ∼ $10^{6}$–$10^{10}$.

It should be noted that the quasi-linear estimates for $\nu{_{iaw}}$ have been found to be ∼ 2–3 orders of magnitude too small in recent Vlasov simulations using realistic mass ratios (Petkaki et al. 2006; Petkaki and Freeman 2008; Yoon and Lui 2006, 2007). Thus, the real scattering rates due to wave-particle interactions could exceed $10^{15}$ times the Coulomb collision rates (i.e., for the slowest rates between ions).

### Derivation of Effective Collisional Operators from Collisionless Fully Kinetic Simulations

Yerger et al. (2025) describes three complementary strategies to obtain effective collision-like operators describing collisionless scattering from simulated data. What sets this work apart from similar papers is that the three strategies are deployed on the same simulation data-set, and therefore their strengths and weaknesses can be directly identified. The dataset used in this work comes from simulations of whistler heat-flux instability, an excellent test case given the dependence of electron scattering patterns on velocity and pitch angle in phase space: the WHFI is mostly oblique and left-hand polarized, hence it resonated with electrons with velocity opposite to the background magnetic field (negative pitch angle). The simulations are PIC simulations run in 2D, since the WHFI propagate at an angle with respect to the background magnetic field. The scattering operators are derived following three approaches: Chapman–Enskog, quasi-linear, Fokker–Planck. In all three cases, a collision-like operator is found, which describes electron pitch-angle scattering by whistler waves. In all cases, an effective collisionality can be identified, which depends on the electron position in phase space (velocity and pitch angle). In principle, these three formulations of the effective scattering term can be completely determined from simulation data. In practice, this proves to be an extremely challenging task. In the Chapman-Enskog derivation, one needs to calculate the derivative in pitch angle of the first order perturbation to the equilibrium velocity distribution function, a forbidding task given the numerical noise associated with PIC simulations. Calculating the effective scattering frequency within the quasi-linear approach requires calculating the Fourier transforms of the magnetic field components. Within the Fokker-Planck approach, the jump moments (i.e., the statistical averages of the changes in particle velocities $\Delta \mathbf{v}$ over a time interval Δt) in pitch angle have to be calculated from particle statistics.

Notwithstanding the differences between the three derivations, certain physically motivated results are consistently recovered. First, the difference in scattering frequency between positive and negative pitch angles, see Eq. (30), is recovered within both the quasi-linear and Fokker-Planck framework (within the Chapman-Enskog derivation, the dependence of the effective scattering on pitch angle is averaged away). Second, the scattering rates scales weakly with the normalized velocity as $\sim (v/ v_{the})^{\alpha}$, with v the velocity and $v_{the}$ the electron thermal velocity, and α different for positive and negative pitch angles.

### Parametrizing Landau Fluid Closures on Collisionless Simulations

The original Hammett-Perkins closure was developed to reproduce kinetic resonances such as Landau damping within a fluid framework in an 1D unmagnetized plasma (Hammett and Perkins 1990). Landau fluid closures have then been extended to higher dimensions and magnetized plasmas (Hunana et al. 2019a). The procedure to develop Landau-fluid closures is described in Hunana et al. (2019a): the perturbed VDF, obtained from the linearized Vlasov equation, is transformed to Fourier space in the gyrotropic limit. The perturbed moments are then calculated by integrating such perturbed distribution function (multiplied by the appropriate power of the velocity) in velocity space. They contain the kinetic plasma response function $R(\zeta )= 1+ \zeta Z(\zeta )$, where $\zeta = \omega /(|k_{par}| v_{th})$, with ω the frequency, $k_{par} $ the parallel wavenumber and $v_{th}$ the thermal velocity, and Z(ζ) the usual plasma dispersion function. To express a given moment through lower moments using only fluid quantities (and therefore to solve the “closure problem”), the kinetic plasma response function is replaced with an approximation using only non-kinetic variables. It is convenient to do so using Padé series, which allows one to approximate a power series as the ratio of two polynomials. The plasma response function R(ζ) in the appropriate limit is then replaced by its n-pole Pade’ expansion $R_{n}(\zeta )$, where n (the number of poles) is the degree of the chosen polynomial in the denominator. The coefficients of the n-pole Padé expansion $R_{n}(\zeta )$ are chosen by matching the asymptotic behavior of R(ζ) in the chosen limit to that of $R_{n}(\zeta )$. Different closures for the same moment can be found using different Padé expansions. These closures, derived in Fourier space, have then to be transformed back to real space.

10-moment codes with Landau fluid (LF) closures have been successfully used for the simulation of tearing instability, plasmoid instability, magnetic reconnection (Ng et al. 2017; Allmann-Rahn et al. 2018; Ng et al. 2020), pressure gradient driven instabilities such as the Lower Hybrid Drift Instability (Ng et al. 2020; Allmann-Rahn et al. 2021), shearing instabilities such as the Kelvin-Helmholtz instability (Lautenbach et al. 2026), decaying turbulence (Lautenbach et al. 2026) and of course temperature anisotropy driven instabilities (Walters et al. 2024), in parameter regimes of relevance for both the terrestrial magnetosphere and the solar wind.

In these cases, LF closures, either non-local (i.e., calculated in Fourier space (Ng et al. 2017)) or local (calculated in real space, following Sharma et al. 2006) are used. In either case, coefficients are present in the heat flux closure. In the case of local closures, these coefficients take the form of an effective wavenumber $k_{0,s}$, with s the particle species, that can be related to characteristic length scales (mainly, skin depth) of the different plasma species. Lautenbach et al. (2026) explores the effects of changing the effective closure parameter $k_{0,s}$ in two-fluid 10-moment simulations with local LF closures of Landau damping, Kelvin-Helmholtz instability and decaying turbulence. The results are compared with reference fully kinetic Vlasov simulations. A very interesting results from this study is that, even if LF closures are derived under the assumption that the plasma is close to Local Thermodynamic Equilibrium, LTE (and hence, that the VDF is essentially a slightly perturbed Maxwellian distribution), they are in fact quite robust with respect to this assumption: two-fluid 10-moment simulations continue to compare well with their fully kinetic reference simulations also quite far from LTE, as long as the $k_{0,s}$ parameter is appropriately chosen.

### Application of Machine Learning to the Closure Problem

A rather recent and continuously growing body of work is applying methods rooted in Machine Learning to the closure problem. Also here, the aim is to reconstruct the heat flux (or its divergence) using first principle simulations as the ground truth.

The closure can either be in symbolic or neural form. In the former case (symbolic closures), the method extracts equations from the data. Examples are Donaghy and Germaschewski (2023) and Cheng et al. (2023). Donaghy and Germaschewski (2023) uses SINDy (Sparse Identification of Non Linear Dynamics (Brunton et al. 2016), already used by Alves and Fiuza (2022) to obtained reduced models from PIC simulations) to obtain symbolic expressions for the six components of the divergence of the electron heat flux from PIC simulations of magnetic reconnection. The discovered closure equations are less sparse that the Landau fluid closure they are compared against, but outperform them for the diagonal pressure components. Cheng et al. (2023), instead, (re-)discover the moment equations up to the pressure equation, including a Landau-fluid-like term for the heat flux closure term, from 1D1V Vlasov simulations using the PDE-Net framework described in Long et al. (2018). In the latter case (neural closures), neural networks are trained, which predict the heat flux closure based on input parameters available from the simulations. In Huang et al. (2025), for example, a ten-moment code is shown to reproduce excellently fully kinetic simulations of linear and non-linear Landau damping when closed with a Fourier Neural Operator (Li et al. 2020) trained on fully kinetic Vlasov simulations of non-linear Landau damping.

In the context of this paper, Bois et al. (2020) is quite interesting. There, a V-net (Milletari et al. 2016) is used to map inputs from a Vlasov–Poisson simulation (density, velocity, electric field, temperature) to the heat flux, in the presence of different levels of collisionality (0.01≤Kn<1). The predicted heat flux is then used to close a fluid model, and results are compared with both fully kinetic and Navier–Stokes simulation results. The fluid model with data driven closure outperforms the Navier–Stokes especially in the low collisionality regime.

## Conclusions and Open Questions

In this article, we review electron transport in collisional and collisionless plasmas, and in the very interesting “in-between” region, when collisional and collisionless processes both participate in the defining of electron transport, see Sect. 3. Electron transport is one of the most relevant cases of electron scale physics having a fundamental impact on the global properties of space and astrophysical systems, see also Artemyev et al. (2026, this collection).

Observations and measurements in many space, astrophysical, and laboratory systems, which we review in Sect. 2, provide both clues to solve the transport puzzle and a number of open questions. Fully kinetic numerical simulations assist in the understanding of these processes. When combined with observational knowledge and theoretical results, these kinetic simulations have the potential to bring large-scale simulations of space and astrophysical plasmas closer to reality (see Sect. 4).

Since a comprehensive transport theory still remains elusive, we identify four central *Challenges* that a successful transport model should be able to address:

- Challenge 1: An improved transport theory should describe how collisional and collisionless processes affect VDFs and, consequently, alter transport properties depending on particle position in phase space. Coulomb collisions affect preferentially low-speed particles, while kinetic processes often dominate over collisions in determining the behaviour of high-speed particles. Effective collision operators must reflect this speed dependence and describe the transition from preferentially collisional to preferentially collisionless regimes of the VDF, see Sect. 3.
- Challenge 2: An improved transport theory should describe the transition from collisional to collisionless transport (and vice versa) within the same system, as a function of the relevant global plasma parameters. This element is essential since many environments of interest are characterized by a change in the transport model as a function of space or time. For example, in the solar wind, the plasma collisionality decreases with heliocentric distance as the solar wind expands from the collisional lower corona into the collisionless heliosphere. Section 3.3 reviews recent progress in this direction.
- Challenge 3: An improved transport theory should describe the effects of turbulence in transport. Turbulence is ubiquitous in space, astrophysical and laboratory plasma, see the Jiang et al. (2026, this collection). The impact of turbulent fluctuations on particle mean free paths and transport is well recognized (e.g., Blasi et al. 2012; Carralero et al. 2021; Coburn et al. 2022), but still not effectively incorporated into transport theory to the point of providing a satisfactory explanation for observations and laboratory measurements. We refer the interested reader to the companion article by Verscharen et al. (2026) for a review of the impact of tangled magnetic fields on electron transport.
- Challenge 4: In parallel with the above-mentioned Challenges, attention should be dedicated to the modeling aspect, given the fundamental and increasing importance of numerical simulations in plasma physics. An effective collision operator is needed that can model the effects of collisional and collisionless scattering processes in large-scale simulations. These types of simulations are computationally too expensive to be run with the inclusion of all small-scale kinetic effects. Such an effective collision operator should depend only on quantities and parameters available in the reduced models that the operator is expected to close. Ideally, this effective, simulation-ready operator will also rise to Challenges 1 to 3. As evident from Sect. 4, significant work has been done to develop an effective operator that can address Challenges 1 and (partially) 3. Relatively little work has been dedicated to developing a simulation-ready operator that can describe the transition between collisional and collisionless systems, with the exception of the very simple electron heat flux closures that asymptotes between Braginskii’s electron conductivity and Hollweg’s free-streaming heat flux.

The solution of these Challenges provides an interpretative key for a number of space and astrophysical processes and open questions (see, e.g., Sect. 2.3 for the so-called cooling flow problem). It also has immediate applications of societal relevance, since controlling particle transport in confinement systems, with increasing temperature and in the presence of turbulence fluctuations, is essential to achieve efficient energy production in fusion devices (Dinklage et al. 2018).

Of the many plasma environments for which transport theories are relevant, one in particular emerges as the ideal playing field for the testing of particle transport models against these four Challenges: the solar wind (Sect. 2.1). In the solar wind, global plasma parameters (including plasma density and plasma-β) change significantly as a function of heliocentric distance and depending on the coronal source regions of the wind. The density decreases with distance which reduces the effect of collisions (Challenge 2). The solar wind presents highly non-Maxwellian electron VDFs, in which a collision-dominated population (the core) coexists with thinner, higher-energy populations (the strahl and the halo). These three populations occupy rather different positions in velocity space (Challenge 1; Feldman et al. 1975; Pilipp et al. 1987; Maksimovic et al. 2005; Štverák et al. 2009; Halekas et al. 2020; Verscharen et al. 2022a). Furthermore, the solar wind is often dubbed a “turbulence laboratory” (Challenge 3; Bruno and Carbone 2013), due to the ubiquituous presence of turbulence and its role in energy transport. Finally, extreme benefits to plasma research and space weather forecasting will follow if simulations were able to transition from fully kinetic to reduced models of solar wind processes, while retaining an accurate description of moment evolution (Challenge 4).

In addition to its scientific relevance, the solar wind is a highly-accessible environment, in which in situ measurements have been collected for decades by a number of missions sampling different heliocentric distances and covering a significant range of parameters (see solar wind observations in Figs. 1, 2, 7, and 9). In contrast to, for instance, laboratory plasmas, the relative size of characteristic electron lengths (Debye length, electron skin depth, electron gyro-radius) compared to the probe (spacecraft) size is such that measurements are not strongly perturbative, so that the electron VDF and its moments can be measured relatively accurately, including the very important heat flux, see Figs. 2 and 9.

The wealth of solar wind observations related to electron transport, VDF relaxation by collisionless and collisional processes, and heat flux regulation has brought to light two specific, transport-related, open science questions that relate to the solar wind and that we add to the Challenges listed above:

- *What are the specific instabilities responsible for heat flux regulation in the solar wind?* In situ observations (Cattell et al. 2021) and fully kinetic simulations run with solar wind parameters (e.g., Kuzichev et al. 2019; Micera et al. 2020, 2021) point to the oblique whistler heat flux instability as the key regulator for strahl heat flux in the solar wind. Moreover, other instabilities in the whistler family are relevant, such as the recently observed (Berčič et al. 2021b) and simulated (Micera et al. 2025) whistler deficit instability. However, the stark reduction in the occurrence of whistler waves at low heliocentric distances (within $\sim 28\; R_{\mathrm {Sun}}$, see Cattell et al. 2022; Choi et al. 2024) point to a less straightforward picture, at least in the young solar wind (Colomban et al. 2024).
- *Does the multi-scale nature of the solar wind influence heat flux regulating instabilities, and hence, particle transport, to a significant degree?* While it is not surprising that multi-scale processes such as turbulence (see Challenge 3) affect particle transport, recent observations (e.g., Nair et al. 2022; Huang et al. 2023) point to a role of meso-scale structures such as switchbacks in heat flux regulation and hence in electron transport in the young solar wind. Switchbacks are magnetic field rotations and local folds of the magnetic field (Kasper et al. 2019; Bale et al. 2019; Raouafi et al. 2023). They are associated with radial plasma jets (Matteini et al. 2014) and hence, potentially, relevant to the acceleration of the solar wind. While they had already been observed by previous missions at larger distances from the Sun (Balogh et al. 1999; Gosling et al. 2009; Horbury et al. 2018), switchbacks occur with unexpectedly high frequency in PSP observations at low heliocentric distances (Pecora et al. 2022; Raouafi et al. 2023; Tenerani et al. 2023). They are characterized by an extreme spread in length, from few tens to $\sim 10^{5}$ ion inertial length (Dudok de Wit et al. 2020; Tenerani et al. 2023). Despite this wide range of sizes, they are definitively large-scale with respect to the heat flux regulating electron scale instabilities, while still quite far from system scales. Recent PSP observations find evidence for whistler wave generation at the magnetic field dips near the edges of switchbacks (Karbashewski et al. 2023; Choi et al. 2024). These waves appear to scatter electrons and hence modify heat flux (Nair et al. 2022; Huang et al. 2023). It is still largely unexplored whether heat flux modifications due to the presence of switchbacks significantly impact overall heat flux evolution.

Electron-mediated heat transport in space, astrophysical, and laboratory plasmas is a very active research field in which theoretical advancements, insights from in situ spacecraft observations, and model development proceed hand-in-hand. In recent years, the field has strongly benefited from electron-scale observations in the solar wind at different heliocentric distances from missions such as Parker Solar Probe and Solar Orbiter. These missions are sampling regions in interplanetary space characterized by a broad range of values of mean free path, Knudsen number, and plasma-β. However, as discussed in Sect. 2.1, multi-point observations of the particles and the electromagnetic fields are essential, even for tasks as simple as identifying the length scale of variation of the electron temperature. The multi-scale nature of all plasma systems and the role of turbulence for particle transport also hint at the essential role of multi-point observations for our understanding of particle transport. Multi-point observations from future missions and mission concepts such as Debye (Wicks et al. 2019), HelioSwarm (Klein et al. 2023), and Plasma Observatory (Retinò et al. 2022; Marcucci and Retino 2024) will therefore prove essential for progress in this important research domain (Verscharen et al. 2022b).

## Footnotes

1 The heat flux and thermal conductivity is similar to Eq. (9), but for an isotropic magnetofluid.

2 The solar-wind experts will have noticed that we have not included datasets recorded by Helios (see Jagarlamudi et al. 2020) and Ulysses (see Gary et al. 1999). We have decide not to include these measurements because the parallel heat flux values are near to the saturation heat flux (Eq. (8)) which is inconsistent with the small currents produced in the solar wind. We look forward to rectifying this situation, but for this reason, we have chose to not include the Ulysses and Helios datasets.

3 here, a better term than ‘collisional frequency’.
